# Supplementary material for: FGF8-mediated gene regulation affects regional identity in human cerebral organoids
Source: eLife. 2024 Nov 1;13:e98096. doi: 10.7554/eLife.98096 (PMC11581432; doi:10.7554/eLife.98096)
Supplement: Figure 3—figure supplement 1—source data 2. — Detailed report on the methods and data for VoxHunt visualization. [file elife-98096-fig3-figsupp1-data2.pdf]

S21306 – Data visualisation using VoxHunt

Matthieu Jung

2 février, 2023

Single-cell dataset used in this report correspond to the A02 dataset. Public Allen Developing Mouse Brain Atlas expression data, stage E13, was used in order to calculate similarity maps between the two dataset using VoxHunt (Fleck et al. 2021) R package version 1.0.1.

Figures 1 to 12 represent visualisations of the results when cells are grouping following Condition and GraphBased clustering.

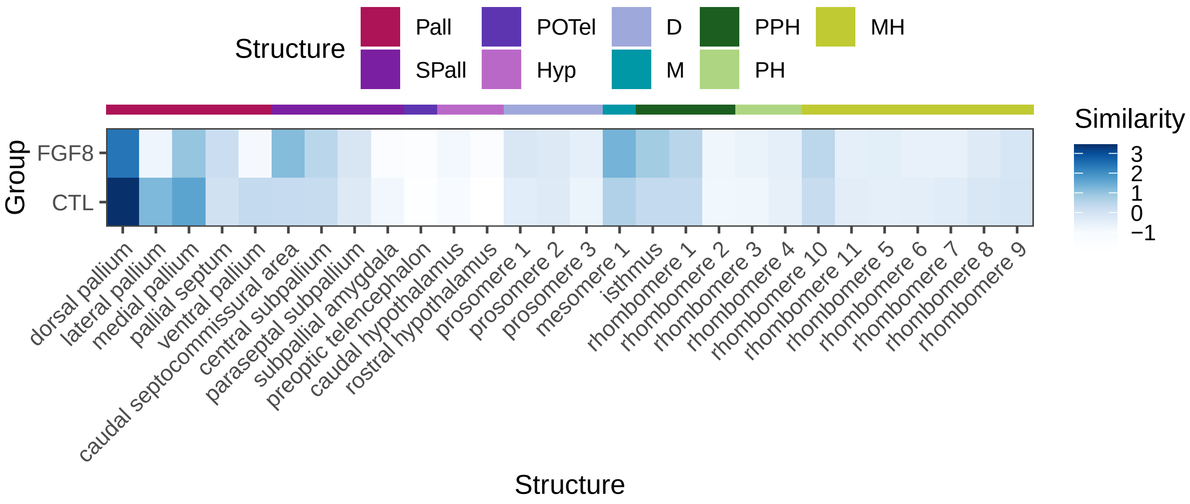

Figure 1: Heatmap of the similarity score between cells grouped with the Condition clustering and cerebral organoids.

Version of R and R packages

```
## R version 4.2.2 (2022-10-31)
## Platform: x86_64-apple-darwin13.4.0 (64-bit)
## Running under: macOS Catalina 10.15.7
##
## Matrix products: default
## BLAS/LAPACK: /Users/jung/bin/miniconda3/envs/rstudio/lib/libopenblasp-r0.3.21.dylib
##
## locale:
## [1] fr_FR.UTF-8/fr_FR.UTF-8/fr_FR.UTF-8/C/fr_FR.UTF-8/fr_FR.UTF-8
##
## attached base packages:
## [1] stats     graphics  grDevices utils     datasets  methods   base
```

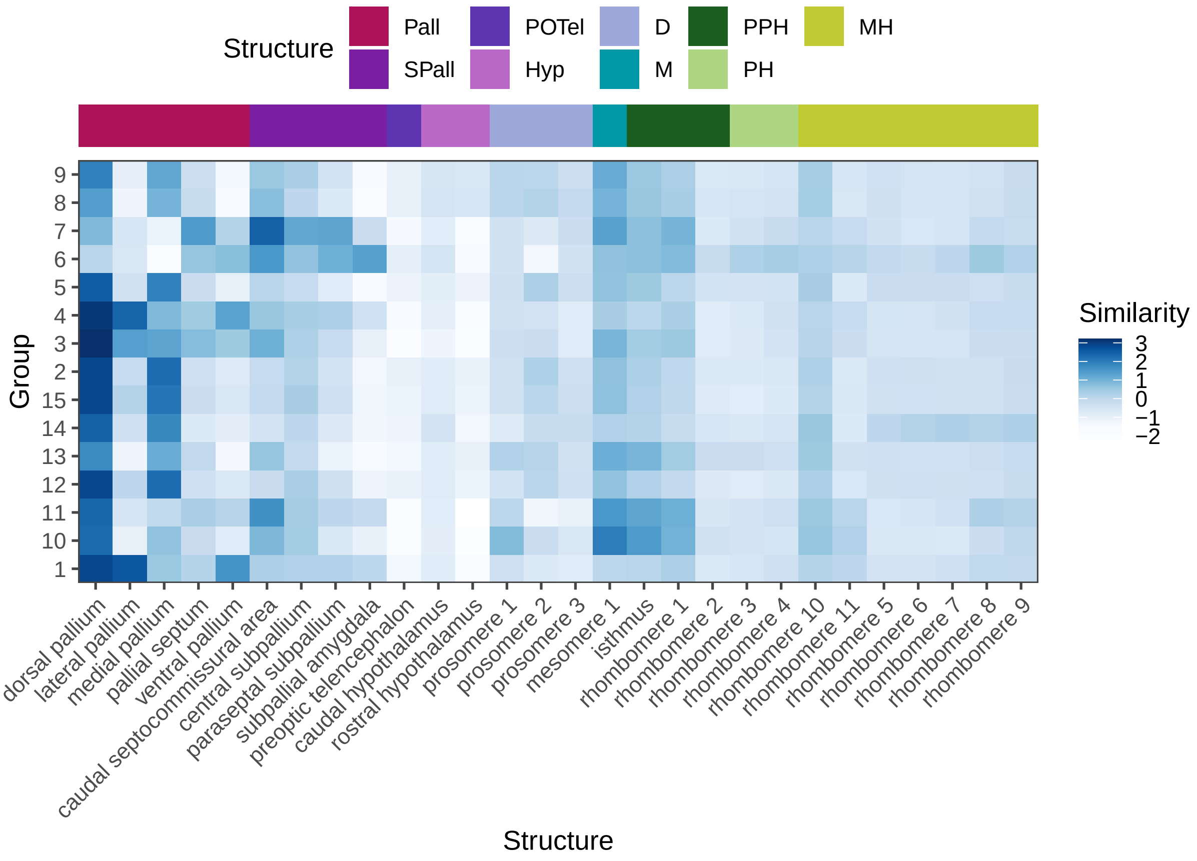

Figure 2: Heatmap of the similarity score between cells grouped with the GraphBased clustering and cerebral organoids.

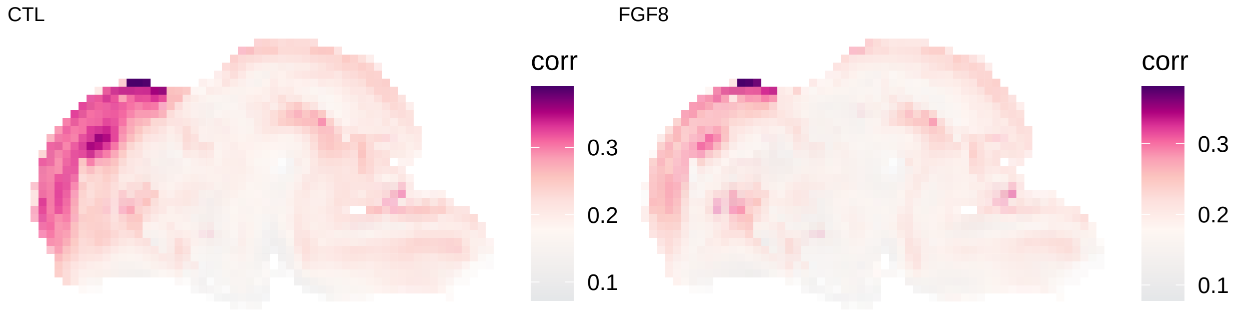

Figure 3: Sagittal view of the similarity score of cells grouped with the Condition clustering.

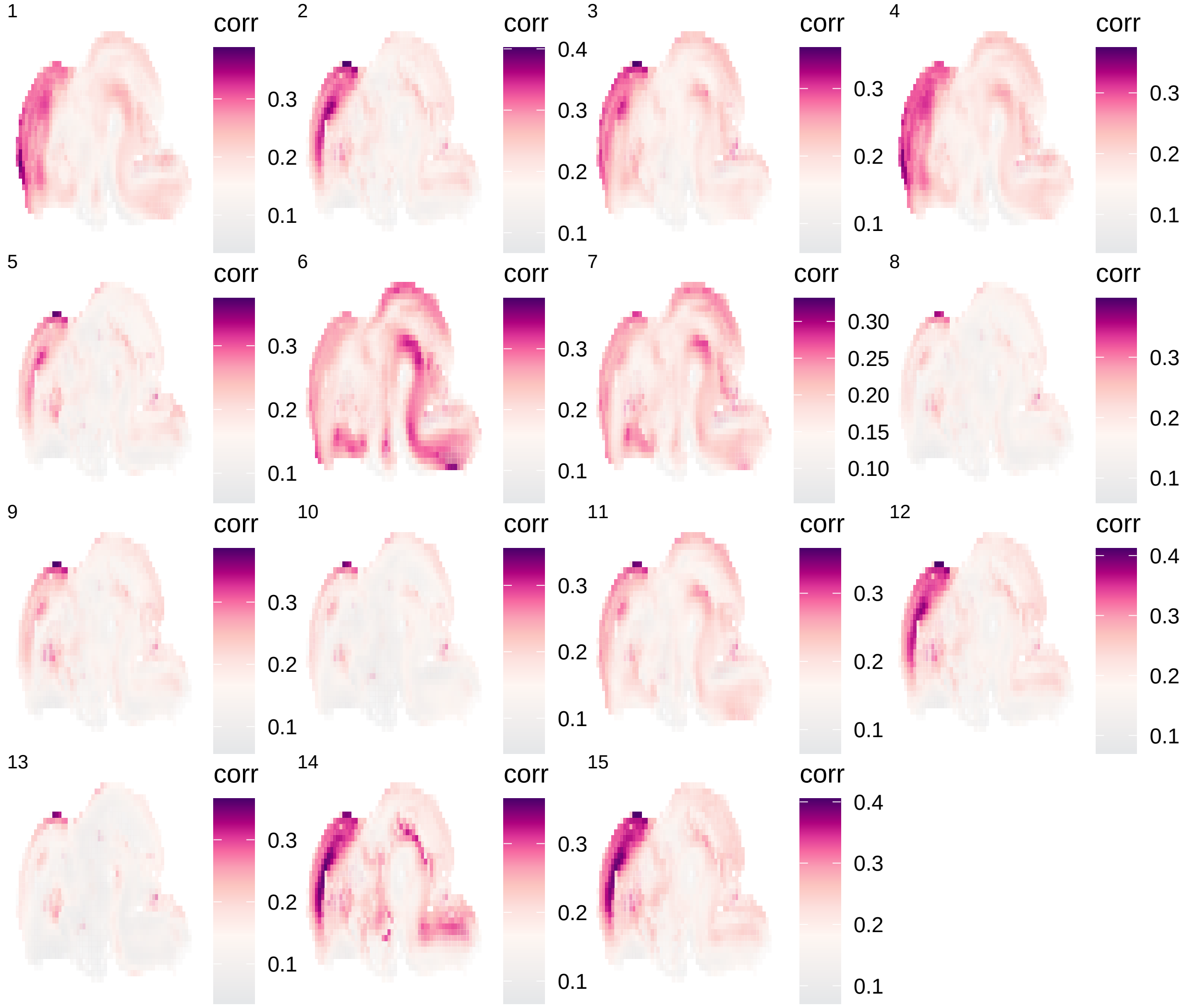

Figure 4: Sagittal view of the similarity score of cells grouped with the GraphBased clustering.

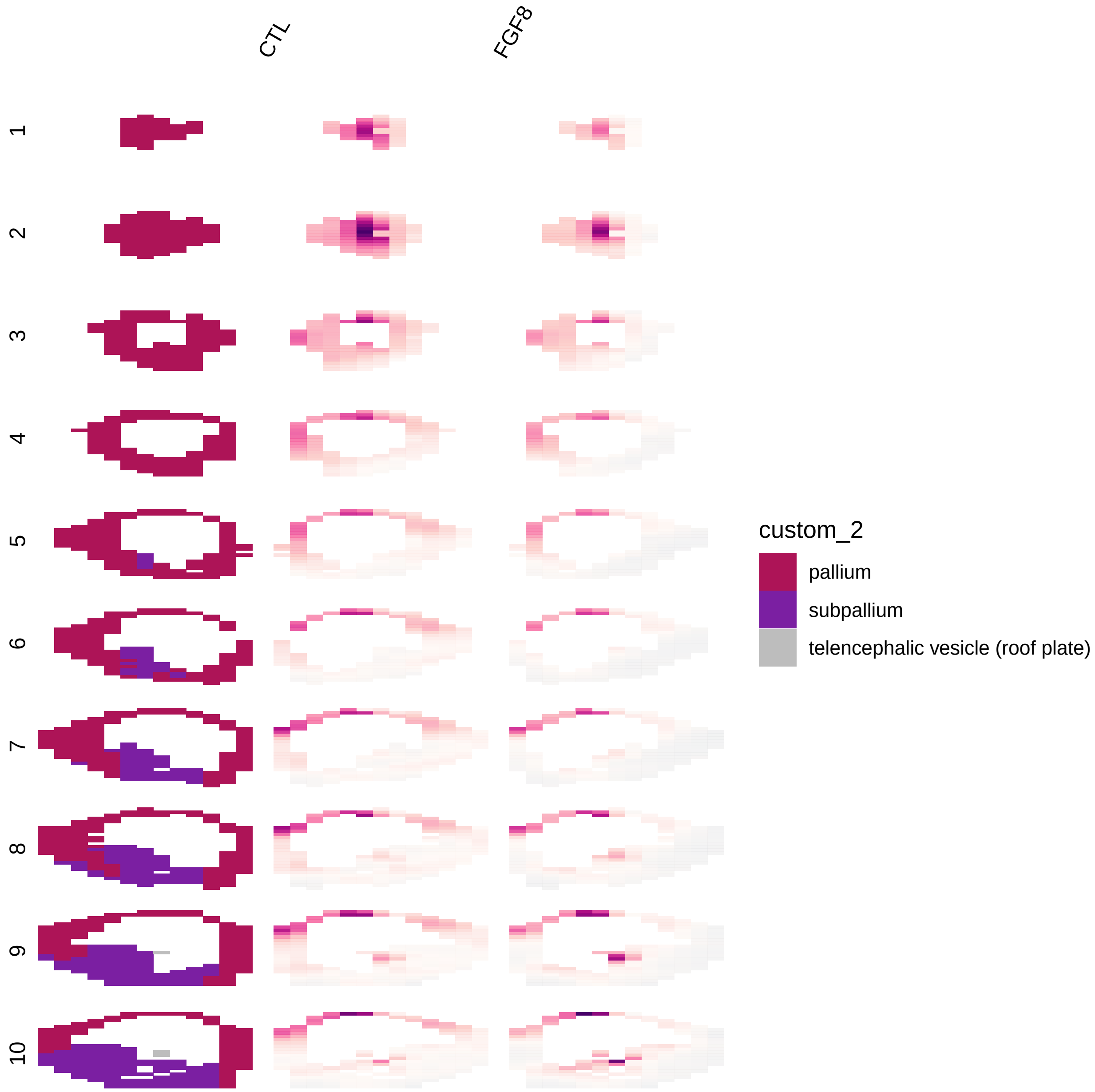

Figure 5: Slice view of slices 1 to 10 and representation the similarity score of cells grouped with the Condition clustering.

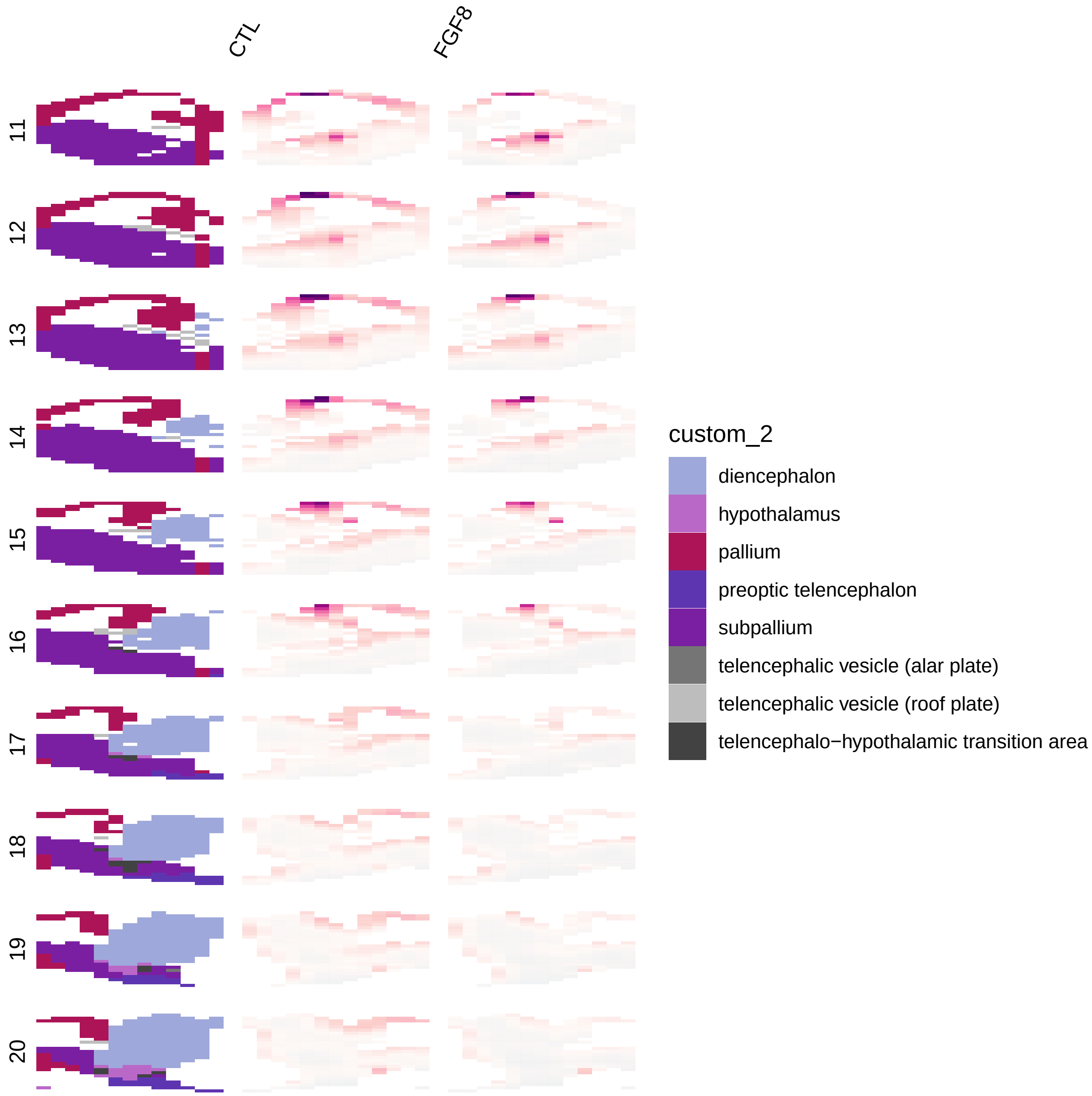

Figure 6: Slice view of slices 11 to 20 and representation the similarity score of cells grouped with the Condition clustering.

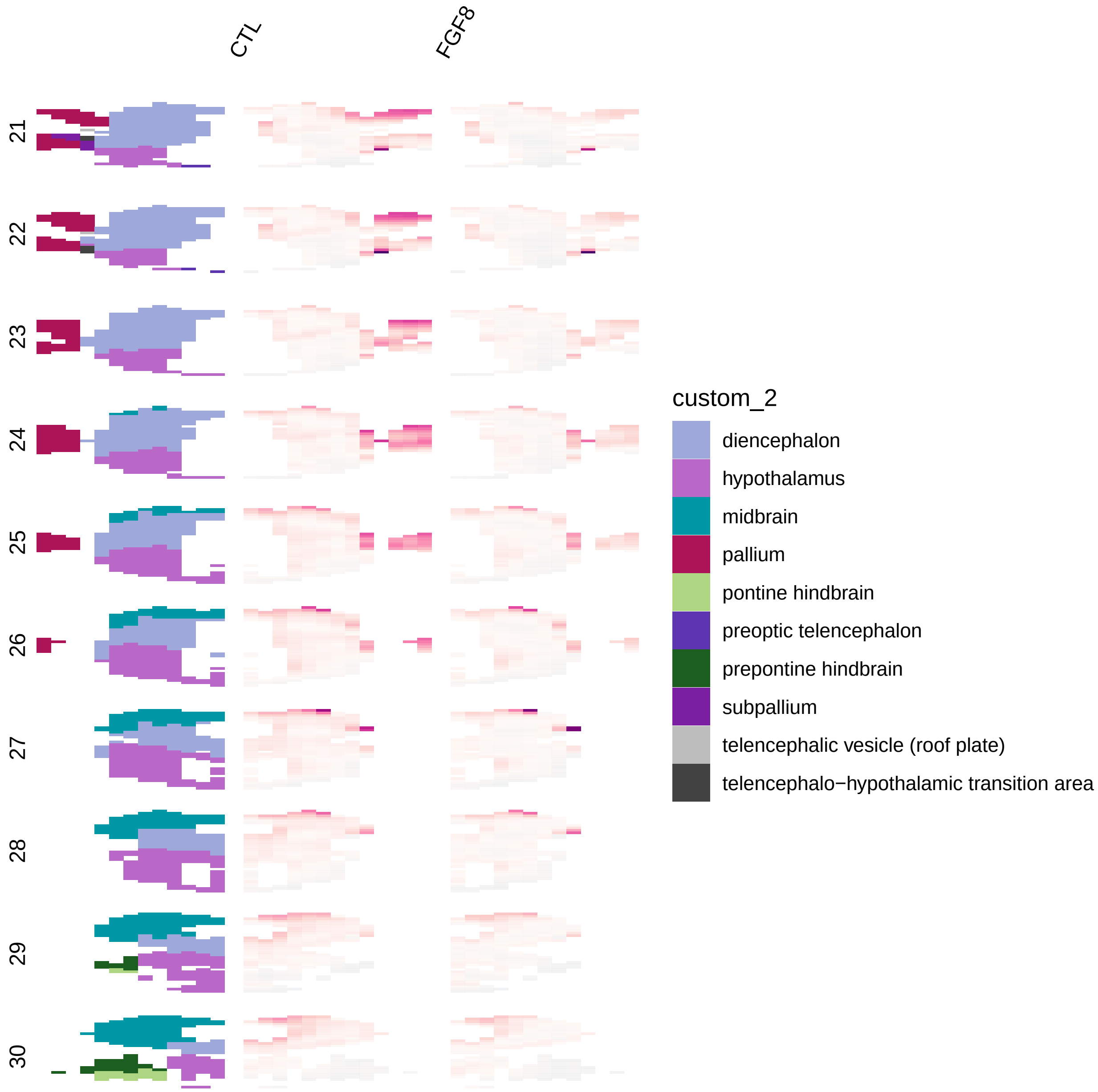

Figure 7: Slice view of slices 21 to 30 and representation the similarity score of cells grouped with the Condition clustering.

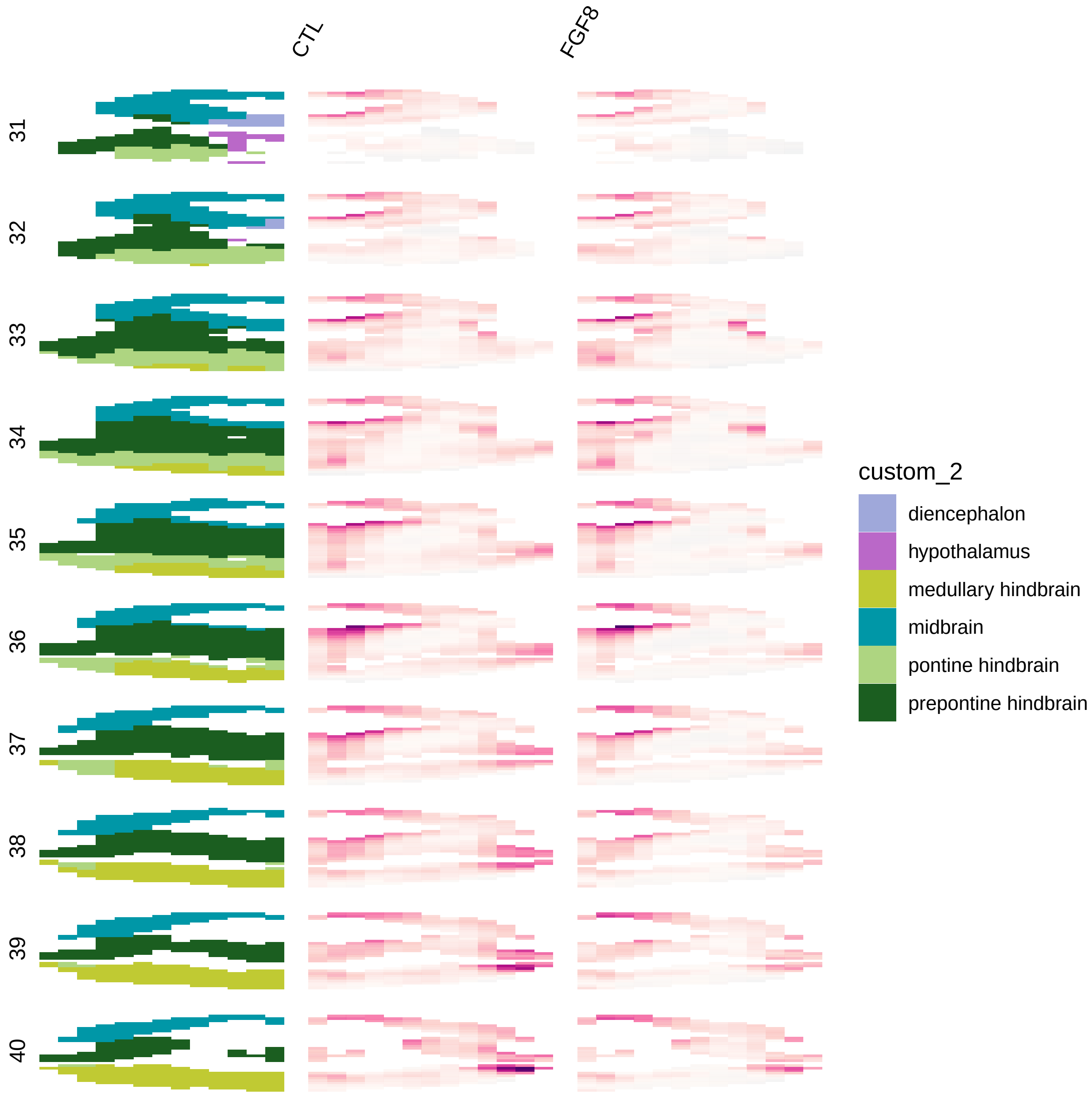

Figure 8: Slice view of slices 31 to 40 and representation the similarity score of cells grouped with the Condition clustering.

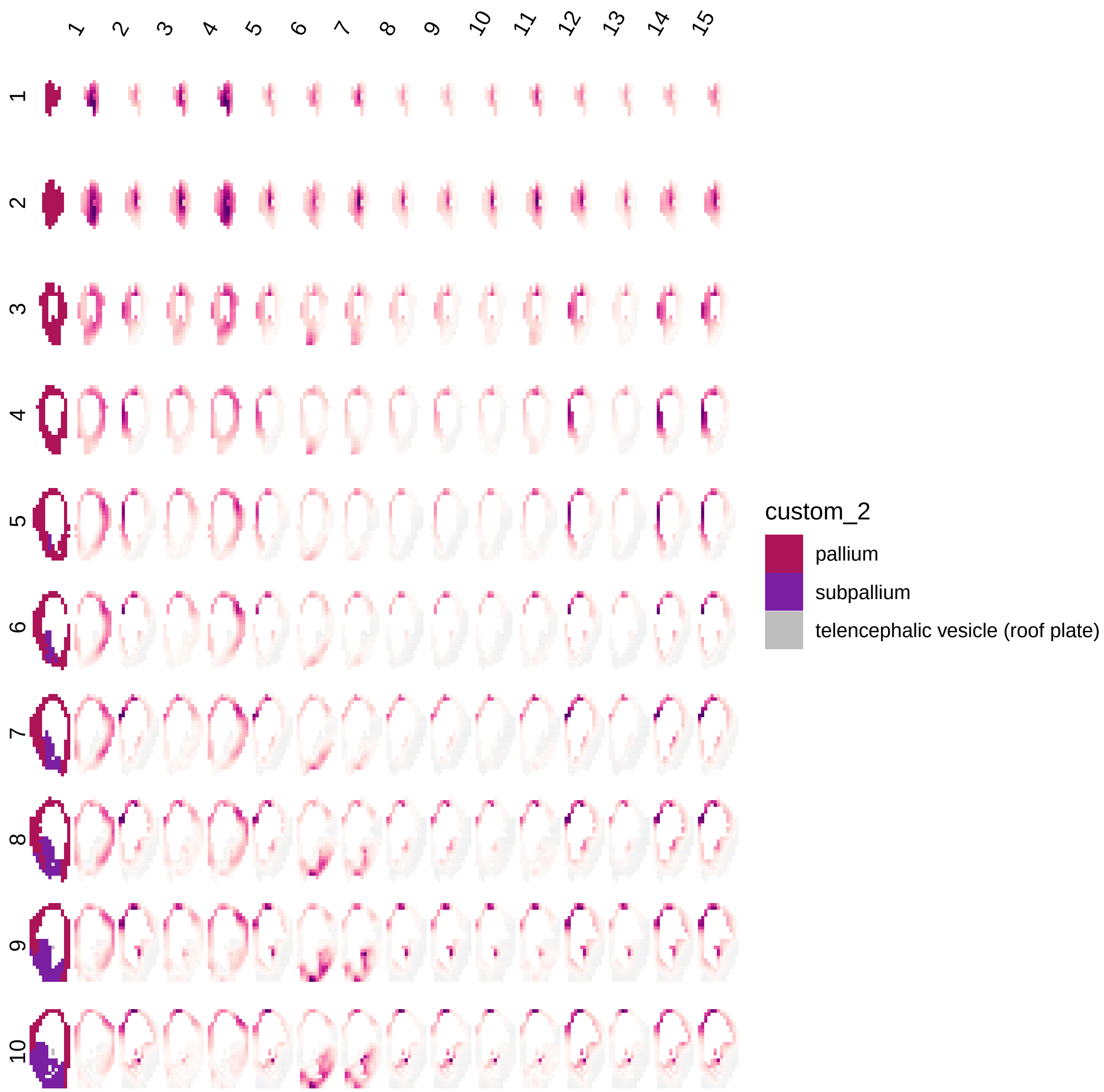

Figure 9: Slice view of slices 1 to 10 and representation the similarity score of cells grouped with the GraphBased clustering.

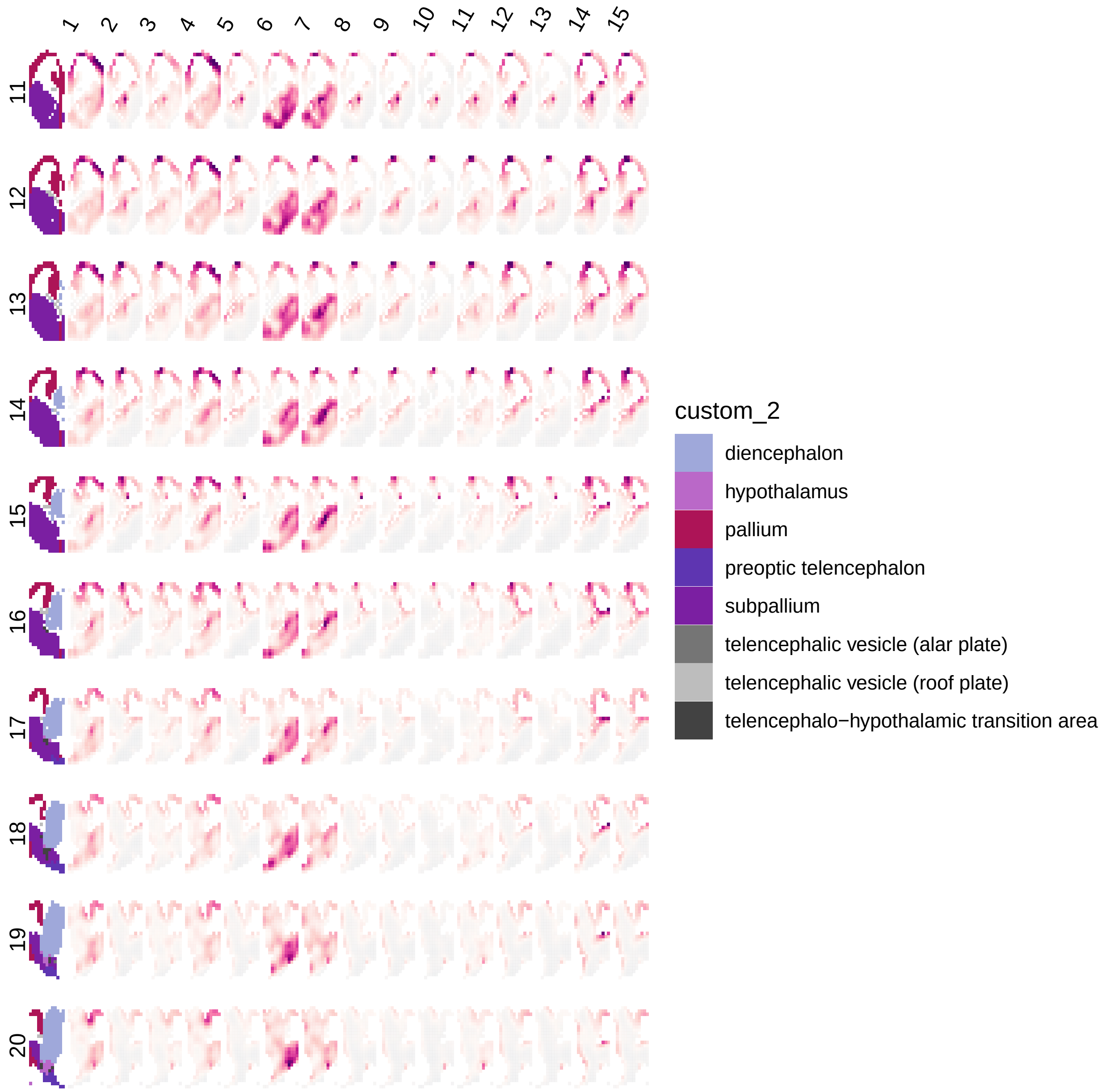

Figure 10: Slice view of slices 11 to 20 and representation the similarity score of cells grouped with the GraphBased clustering.

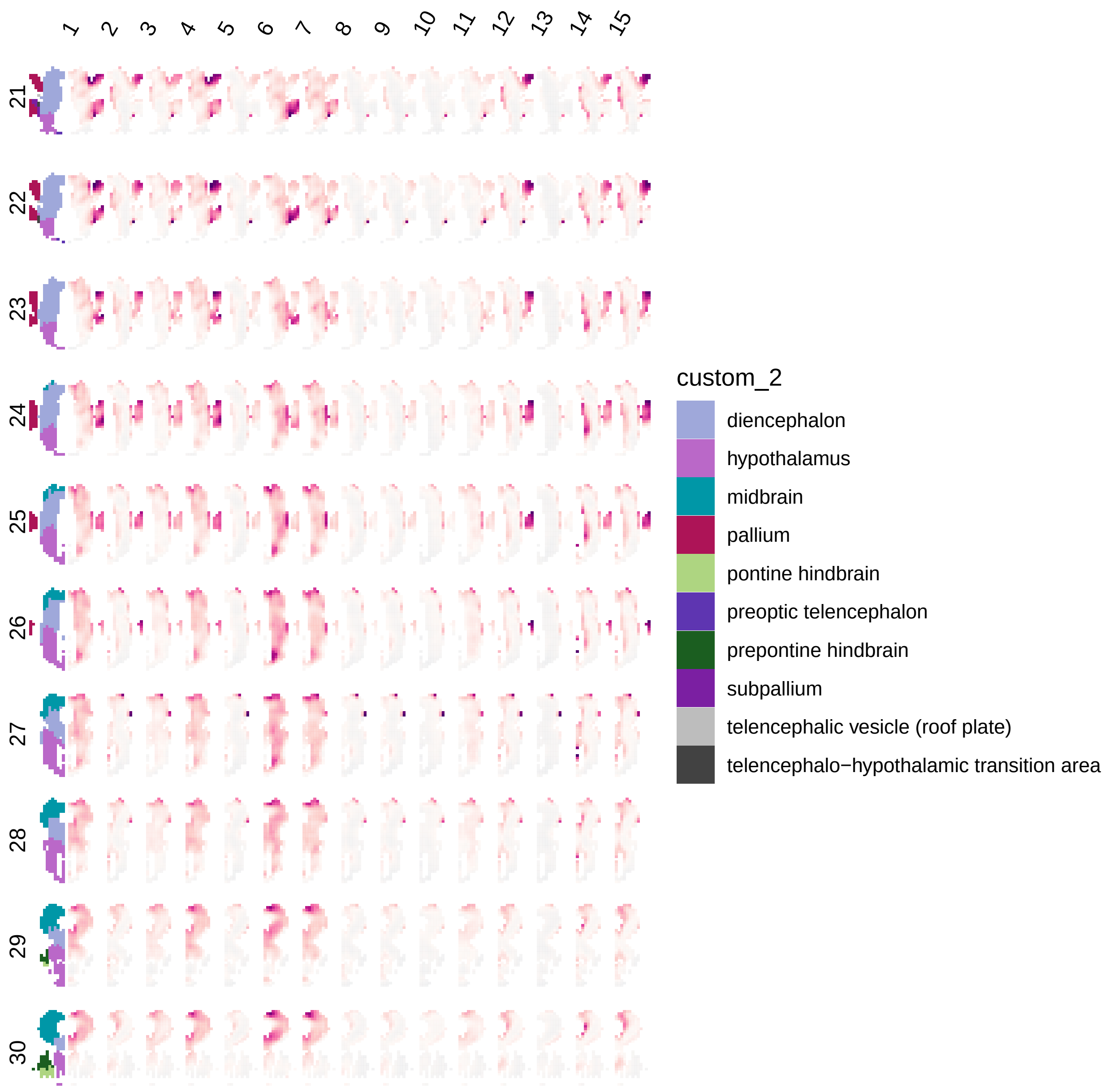

Figure 11: Slice view of slices 21 to 30 and representation the similarity score of cells grouped with the GraphBased clustering.

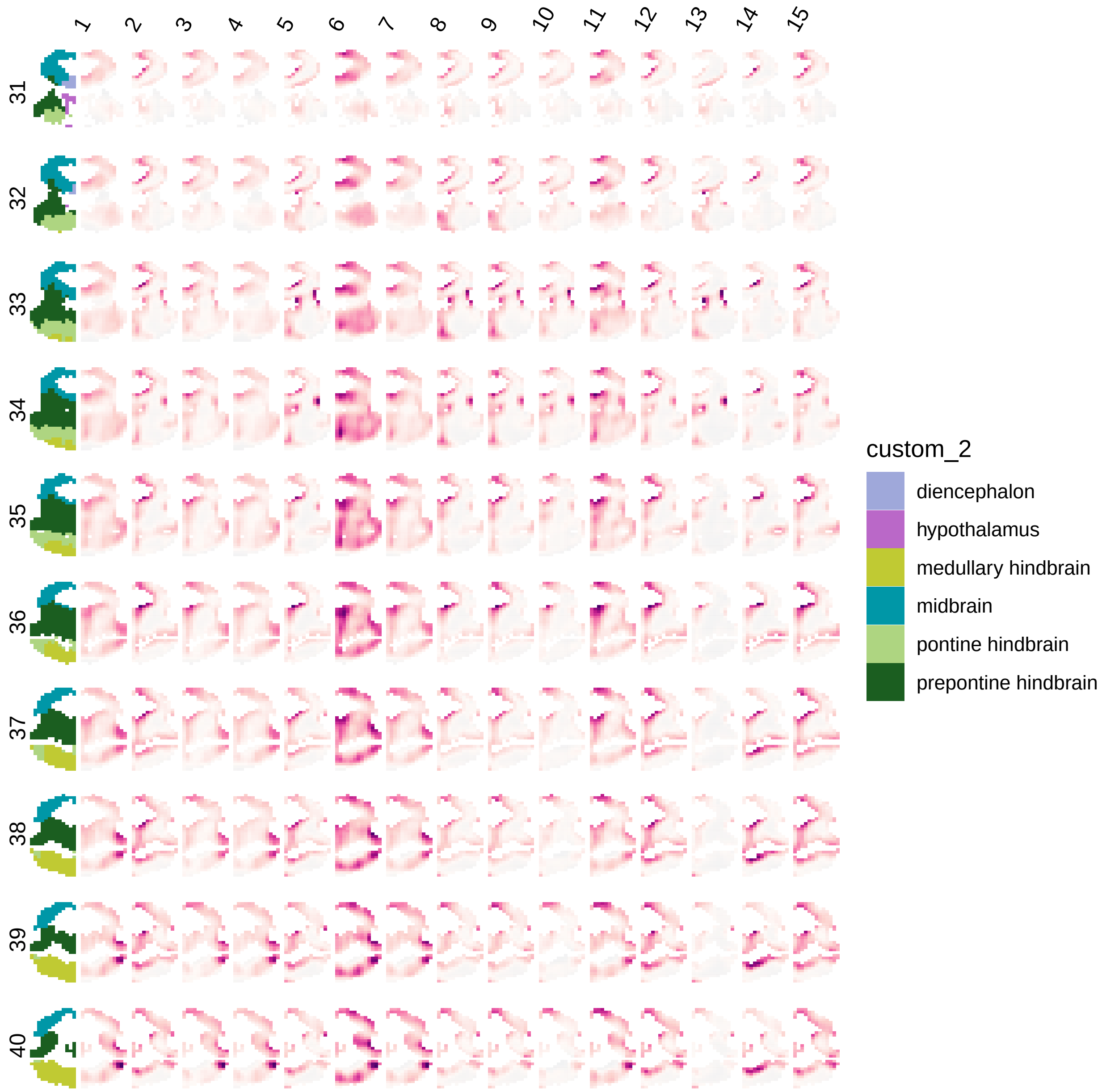

Figure 12: Slice view of slices 31 to 40 and representation the similarity score of cells grouped with the GraphBased clustering.

```
##
## other attached packages:
## [1] SeuratObject_4.1.3 Seurat_4.3.0       voxhunt_1.0.1
##
## loaded via a namespace (and not attached):
##   [1] Rtsne_0.16             colorspace_2.0-3       deldir_1.0-6
##   [4] ellipsis_0.3.2         ggridges_0.5.4         rstudioapi_0.14
##   [7] spatstat.data_3.0-0    farver_2.1.1           leiden_0.4.3
##  [10] listenv_0.8.0          ggrepel_0.9.2          fansi_1.0.3
##  [13] codetools_0.2-18       splines_4.2.2          knitr_1.41
##  [16] polyclip_1.10-4        jsonlite_1.8.4         ica_1.0-3
##  [19] cluster_2.1.4          png_0.1-8              uwot_0.1.14
##  [22] shiny_1.7.3            sctransform_0.3.5      spatstat.sparse_3.0-0
##  [25] compiler_4.2.2         httr_1.4.4             assertthat_0.2.1
##  [28] Matrix_1.5-3           fastmap_1.1.0          lazyeval_0.2.2
##  [31] cli_3.4.1              later_1.3.0            htmltools_0.5.4
##  [34] tools_4.2.2            igraph_1.3.5           gtable_0.3.1
##  [37] glue_1.6.2             RANN_2.6.1             reshape2_1.4.4
##  [40] dplyr_1.0.10           Rcpp_1.0.9             scattermore_0.8
##  [43] vctrs_0.5.1            nlme_3.1-160           spatstat.explore_3.0-5
##  [46] progressr_0.11.0       lmtest_0.9-40          spatstat.random_3.0-1
##  [49] xfun_0.35              stringr_1.5.0          globals_0.16.2
##  [52] mime_0.12              miniUI_0.1.1.1         lifecycle_1.0.3
##  [55] irlba_2.3.5.1          goftest_1.2-3          future_1.29.0
##  [58] MASS_7.3-58.1          zoo_1.8-11             scales_1.2.1
##  [61] promises_1.2.0.1       spatstat.utils_3.0-1   parallel_4.2.2
##  [64] RColorBrewer_1.1-3     yaml_2.3.6             reticulate_1.26
##  [67] pbapply_1.6-0          gridExtra_2.3          ggplot2_3.4.0
##  [70] stringi_1.7.8          rlang_1.0.6            pkgconfig_2.0.3
##  [73] matrixStats_0.63.0     evaluate_0.18          lattice_0.20-45
##  [76] ROCR_1.0-11            purrr_0.3.5            tensor_1.5
##  [79] labeling_0.4.2         patchwork_1.1.2        htmlwidgets_1.5.4
##  [82] cowplot_1.1.1          tidyselect_1.2.0       parallelly_1.32.1
##  [85] RcppAnnoy_0.0.20       plyr_1.8.8             magrittr_2.0.3
##  [88] R6_2.5.1               generics_0.1.3         DBI_1.1.3
##  [91] withr_2.5.0            pillar_1.8.1           fitdistrplus_1.1-8
##  [94] survival_3.4-0         abind_1.4-5            sp_1.5-1
##  [97] tibble_3.1.8           future.apply_1.10.0    KernSmooth_2.23-20
## [100] utf8_1.2.2             spatstat.geom_3.0-3    plotly_4.10.1
## [103] rmarkdown_2.18         grid_4.2.2             data.table_1.14.6
## [106] digest_0.6.30          xtable_1.8-4           tidyr_1.2.1
## [109] httpuv_1.6.6           munsell_0.5.0          viridisLite_0.4.1
## [112] egg_0.4.5
```

References

Fleck, Jonas Simon, Fátima Sanchís-Calleja, Zhisong He, Margozata Santel, Michael James Boyle, J. Gray Camp, and Barbara Treutlein. 2021. "Resolving Organoid Brain Region Identities by Mapping Single-Cell Genomic Data to Reference Atlases." *Cell Stem Cell*. https://doi.org/10.1016/j.stem.2021.02.015.
