# Supplementary material for: FGF8-mediated gene regulation affects regional identity in human cerebral organoids
Source: eLife. 2024 Nov 1;13:e98096. doi: 10.7554/eLife.98096 (PMC11581432; doi:10.7554/eLife.98096)
Supplement: Figure 3—figure supplement 1—source data 4. — Detailed methods and data report for the trajectory analysis of glutamatergic progenitors and neurons (clusters 1, 2, 3, 4, 5, 12, 13, 14, and 15). [file elife-98096-fig3-figsupp1-data4.pdf]

S21306 – Trajectory analysis: CTL drosal cells

Matthieu Jung

16 September, 2022

Data used for trajectory inference

NULL Input data correspond to the 10X Genomics A02 data.

Only CTL cells belonging to cluster 1, 2, 3, 4, 5, 12, 13, 14 or 15 have been kept for further analysis.

Data were normalized with the log normalize method before inferring trajectory.

Only genes with more than 5 UMI count(s) detected in at least 10 cell(s) have been kept for the analysis.

Final data used in this analysis contain **8246 cells** and **5827 genes**.

Cells are distributed as follows:

Cluster	Number of cells
1	1997
2	1112
3	1106
4	1022
5	737
12	648
13	561
14	568
15	495

Trajectory inference

Trajectory inference was performed using slingshot method (K et al. 2018) implemented in the `dyno` package (W et al. 2019).

Following figures represent the inferred trajectory in different situation as described in their legends.

Trajectory differential expression

The goal is to estimate genes where the expression changes in cells at a given milestone. Following figure provide a global overview of the 100 most predictive genes that change anywhere in the trajectory.

Following figures represent heatmaps of genes with an expression changing in cells at a given milestone and the inferred trajectory of the first four genes for each milestone.

The file *feature_importances.tsv* provides the gene overall importance and importance at each milestone.

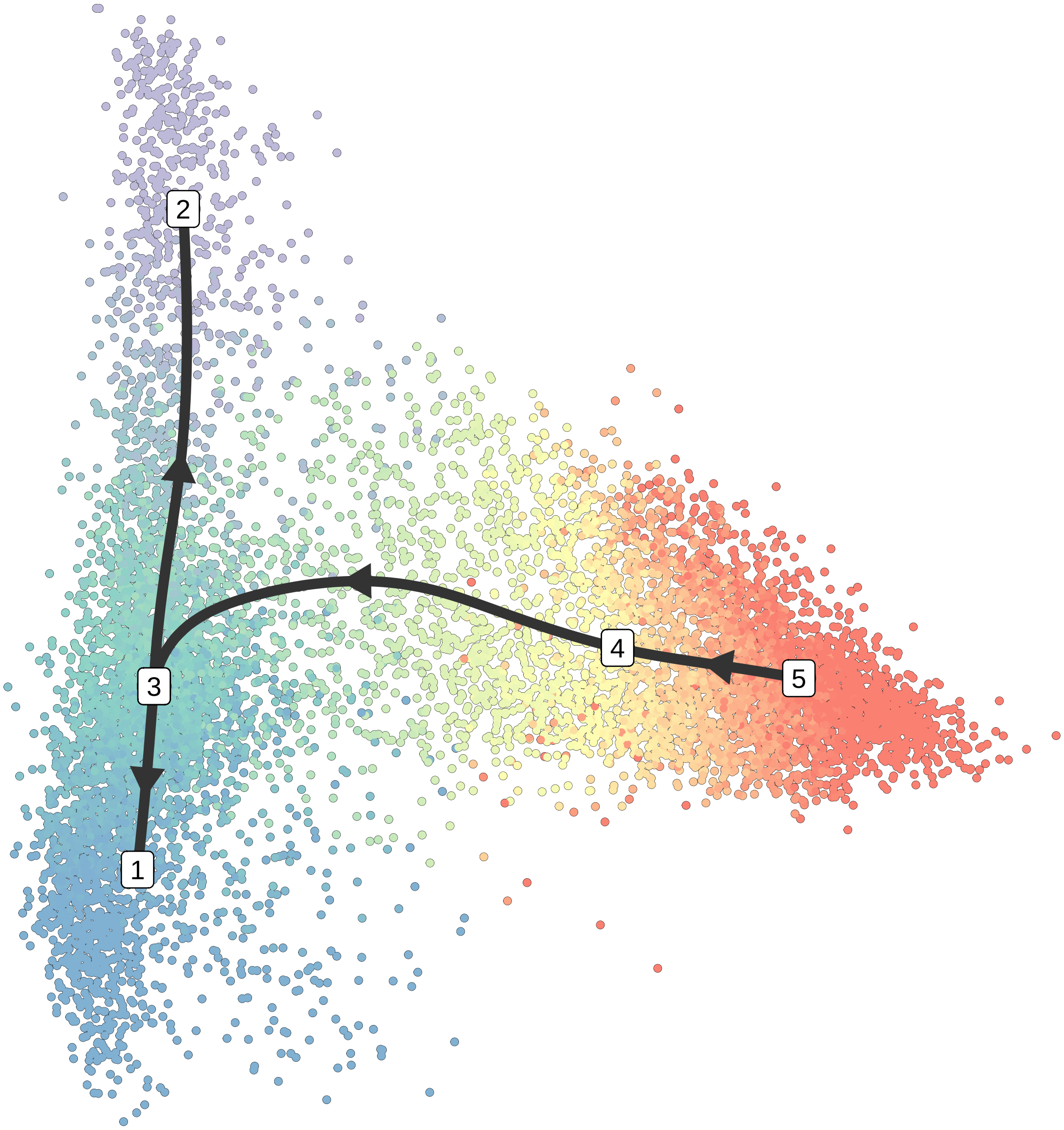

Figure 1: Trajectory colored by cell ordering in which every milestone gets a color and the color changes gradually between the milestones.

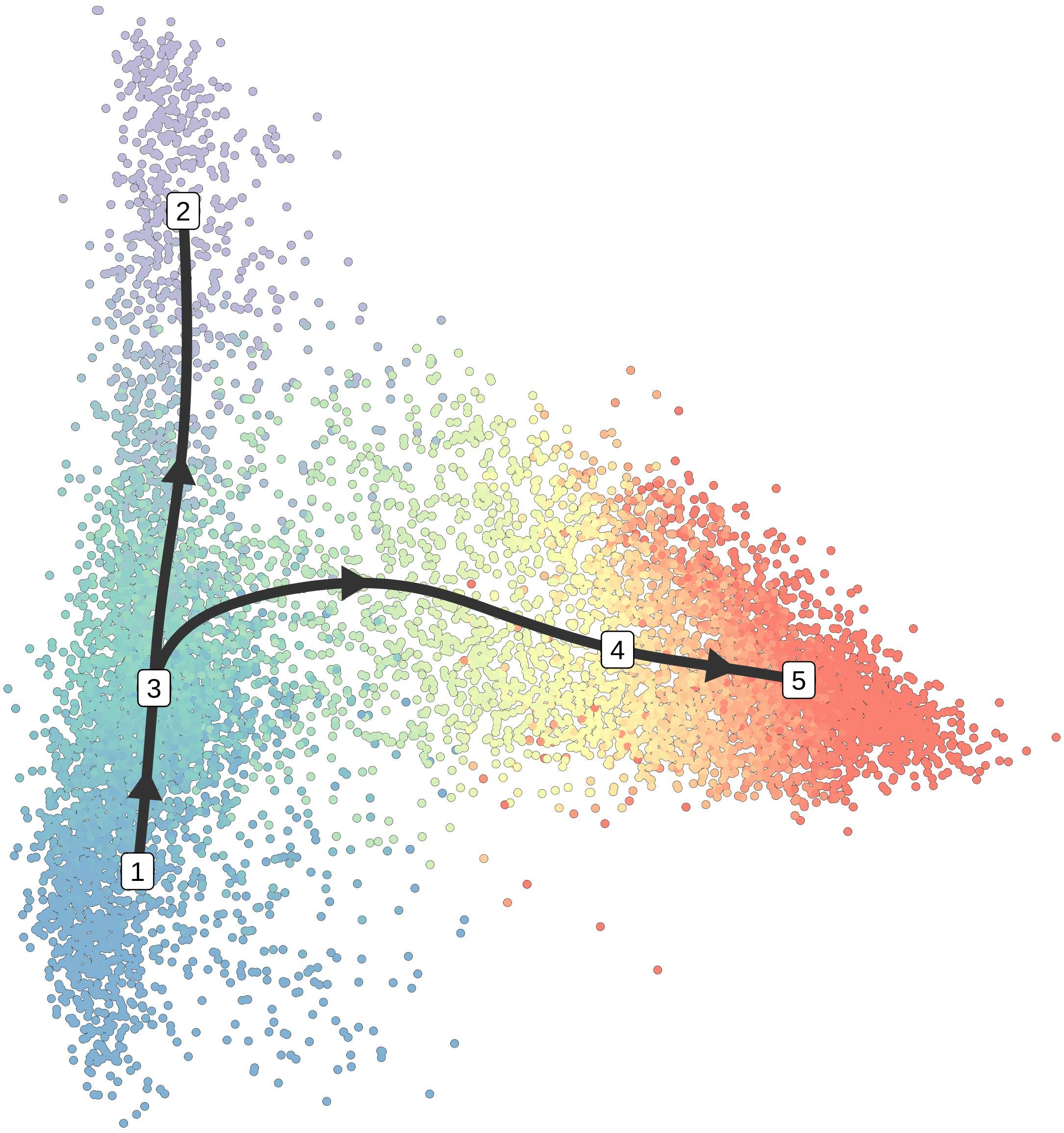

Figure 2: Trajectory colored by cell ordering after rooting. Chosen root correspond to the milestone labelled ‘1‘.

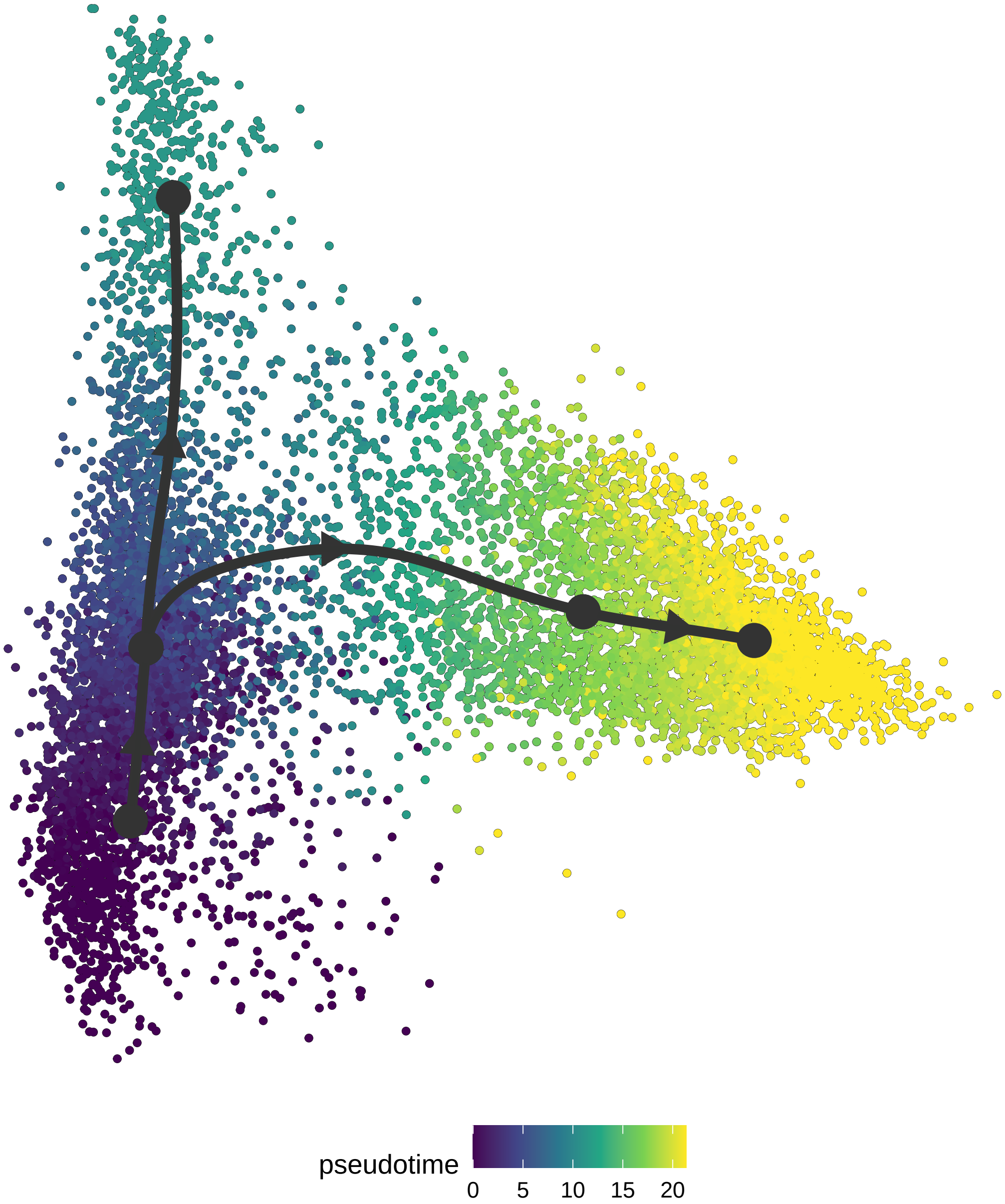

Figure 3: Trajectory colored by pseudotime (the distance to the root milestone).

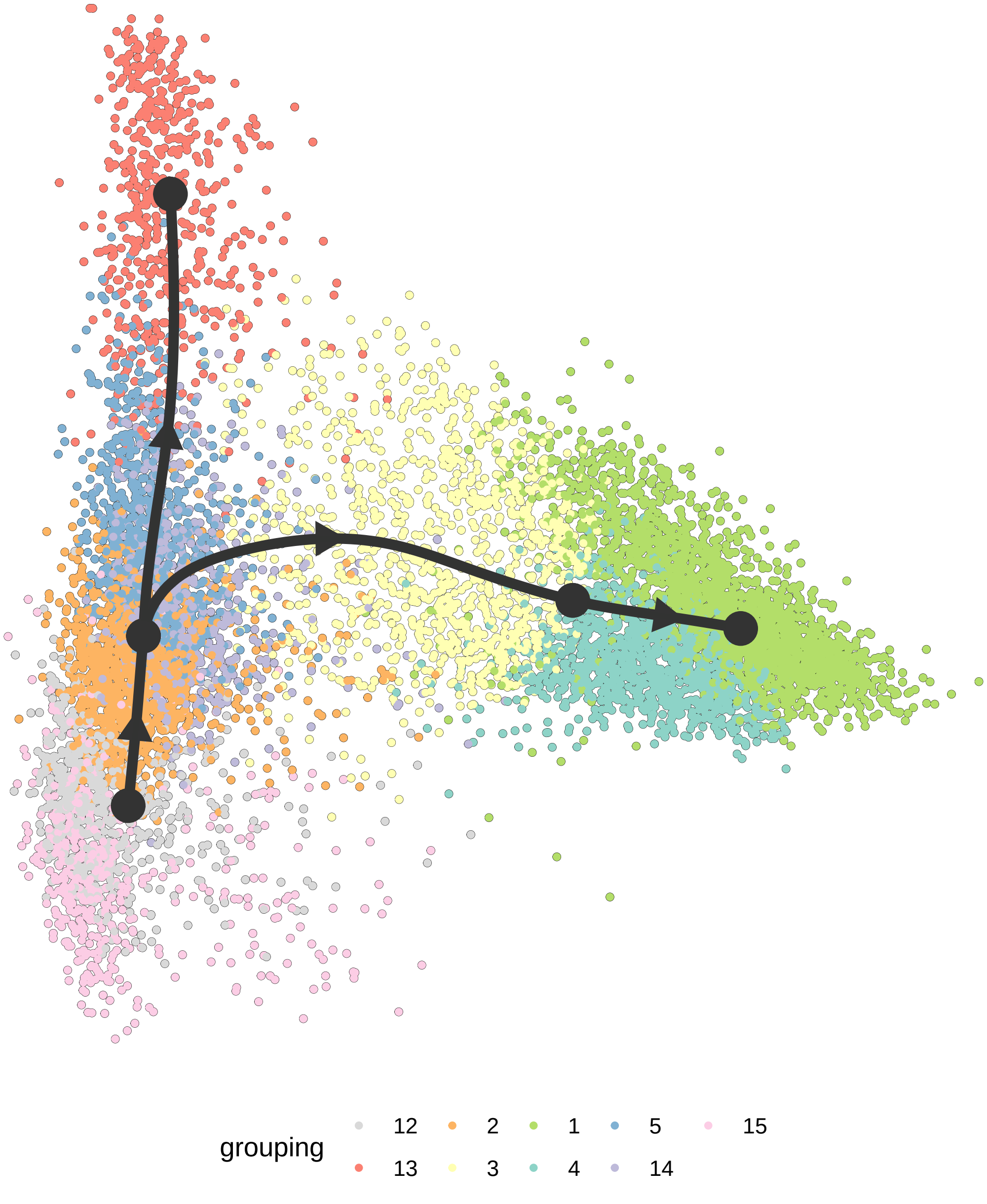

Figure 4: Trajectory colored by cell cluster

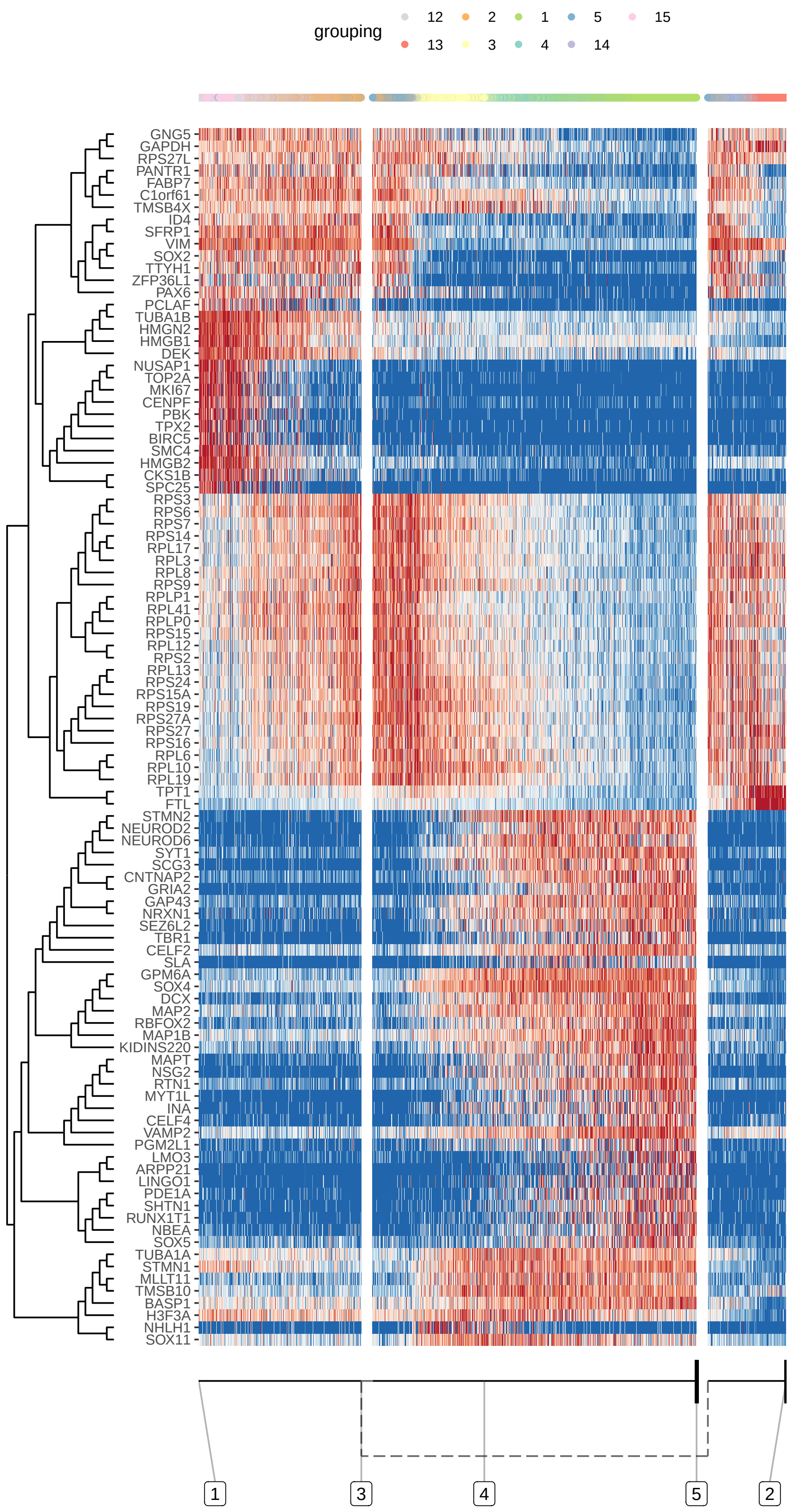

Figure 5: Heatmap representing the 100 most predictive genes that change anywhere in the trajectory.

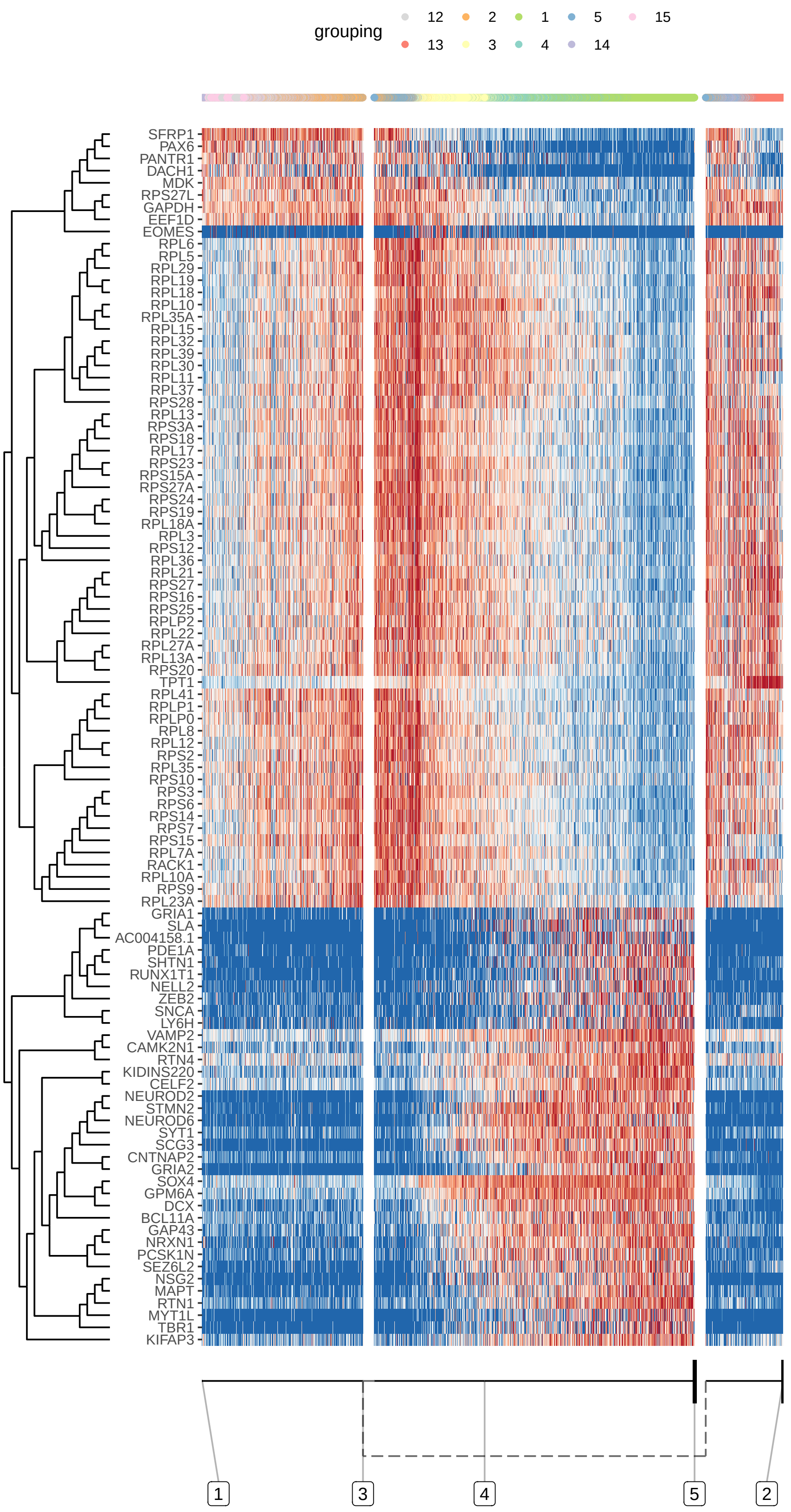

Figure 6: Heatmap representing 100 genes with an expression changing at milestone labelled '4'.

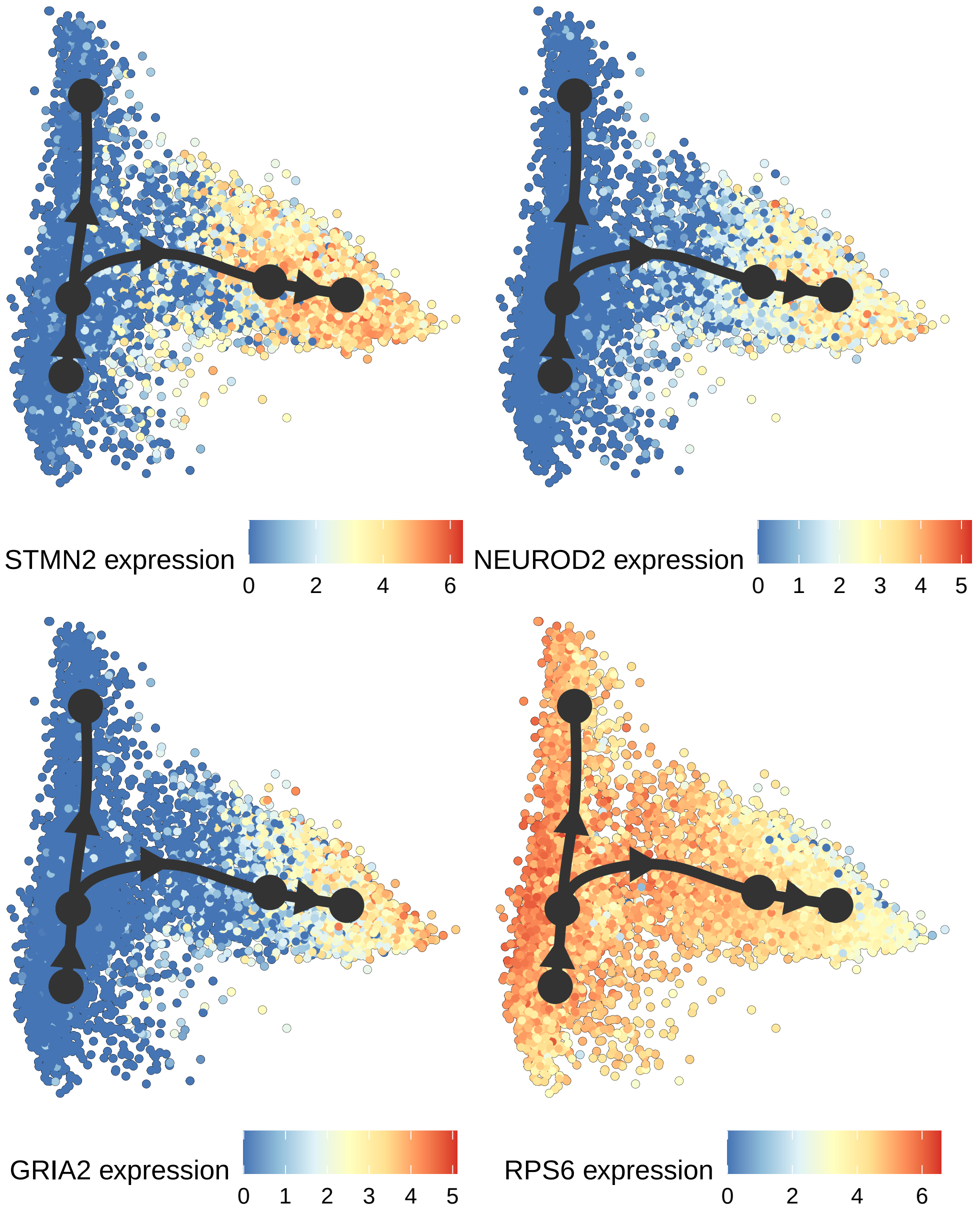

Figure 7: Four first genes with an expression changing at milestone labelled ‘4‘ represented on the infered trajectory.

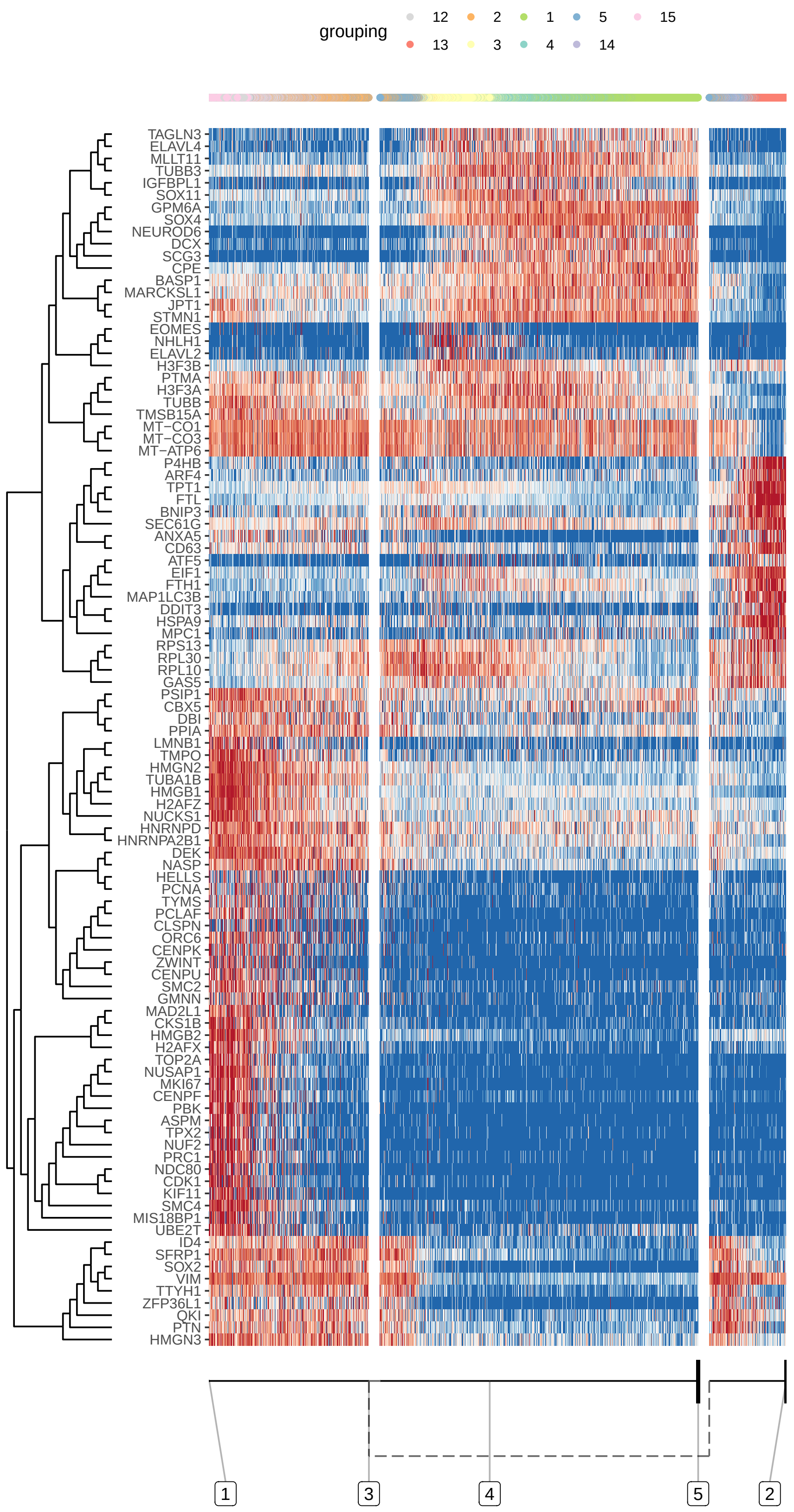

Figure 8: Heatmap representing 100 genes with an expression changing at milestone labelled '3'.

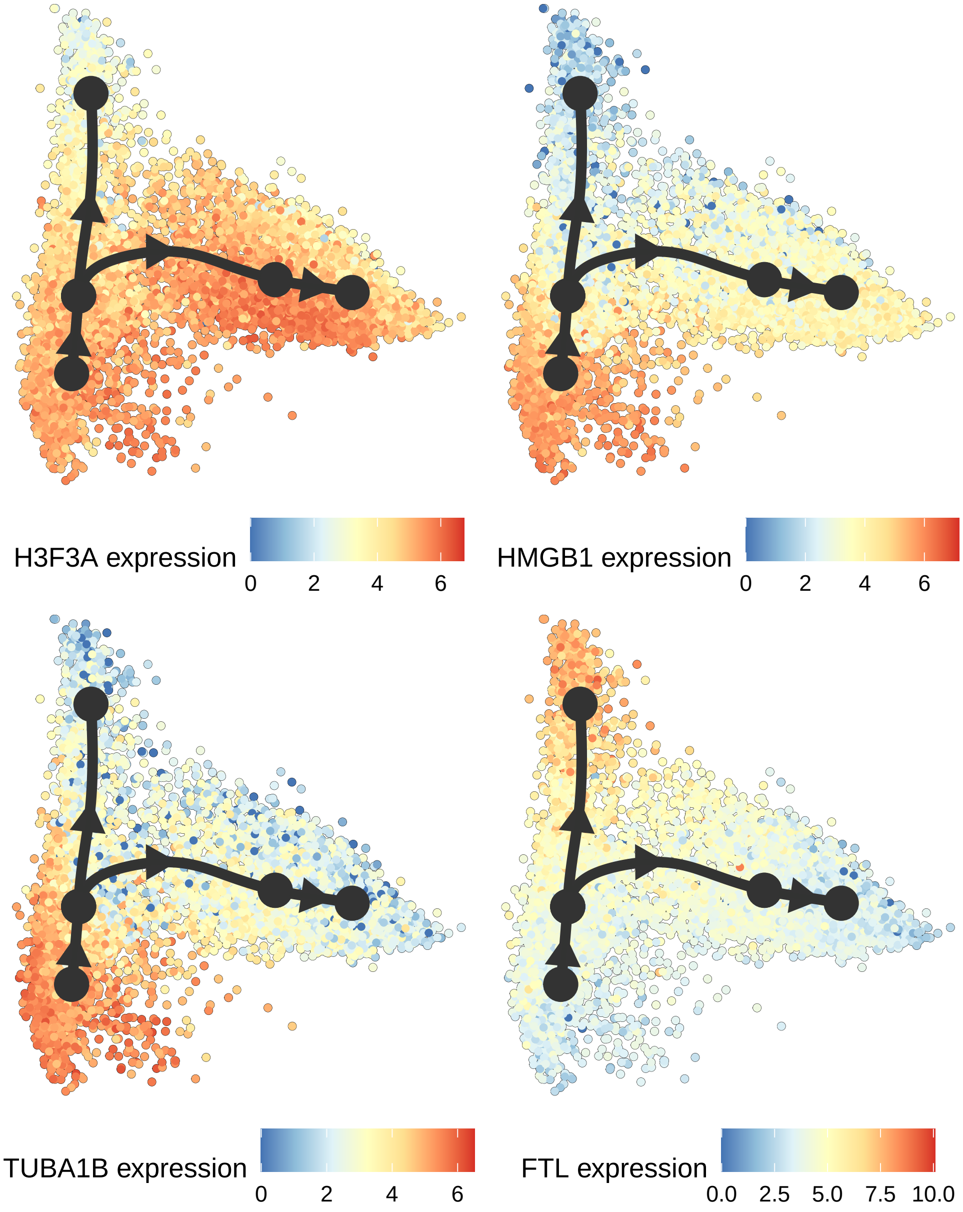

Figure 9: Four first genes with an expression changing at milestone labelled ‘3‘ represented on the infered trajectory.

Version of R and R packages

```
## R version 4.1.1 (2021-08-10)
## Platform: x86_64-conda-linux-gnu (64-bit)
## Running under: Debian GNU/Linux 11 (bullseye)
##
## Matrix products: default
## BLAS/LAPACK: /opt/conda/envs/r411/lib/libopenblasp-r0.3.15.so
##
## locale:
##  [1] LC_CTYPE=C.UTF-8       LC_NUMERIC=C           LC_TIME=C.UTF-8
##  [4] LC_COLLATE=C.UTF-8     LC_MONETARY=C.UTF-8    LC_MESSAGES=C.UTF-8
##  [7] LC_PAPER=C.UTF-8       LC_NAME=C              LC_ADDRESS=C
## [10] LC_TELEPHONE=C         LC_MEASUREMENT=C.UTF-8 LC_IDENTIFICATION=C
##
## attached base packages:
## [1] stats     graphics  grDevices utils     datasets  methods   base
##
## other attached packages:
##  [1] SeuratObject_4.0.2  Seurat_4.0.5        forcats_0.5.1
##  [4] stringr_1.4.0       dplyr_1.0.6         purrr_0.3.4
##  [7] readr_1.4.0         tidyr_1.1.3         tibble_3.1.2
## [10] ggplot2_3.3.5       tidyverse_1.3.1     dyno_0.1.2
## [13] dynwrap_1.2.2       dynplot_1.1.2       dynmethods_1.0.5
## [16] dynguidelines_1.0.1 dynfeature_1.0.0
##
## loaded via a namespace (and not attached):
##   [1] utf8_1.2.2            reticulate_1.20       tidyselect_1.1.1
##   [4] htmlwidgets_1.5.3     grid_4.1.1            ranger_0.13.1
##   [7] Rtsne_0.15            munsell_0.5.0         codetools_0.2-18
##  [10] ica_1.0-2             future_1.21.0         miniUI_0.1.1.1
##  [13] withr_2.5.0           colorspace_2.0-1      highr_0.9
##  [16] knitr_1.35            rstudioapi_0.13       ROCR_1.0-11
##  [19] tensor_1.5            listenv_0.8.0         labeling_0.4.2
##  [22] polyclip_1.10-0       bit64_4.0.5           farver_2.1.0
##  [25] rprojroot_2.0.2       parallelly_1.25.0     vctrs_0.3.8
##  [28] generics_0.1.0        xfun_0.28             R6_2.5.1
##  [31] GA_3.2.2              graphlayouts_0.8.0    hdf5r_1.3.5
##  [34] spatstat.utils_2.1-0  assertthat_0.2.1      promises_1.2.0.1
##  [37] scales_1.1.1          ggraph_2.0.5          lmds_0.1.0
##  [40] gtable_0.3.0          babelwhale_1.0.3      globals_0.14.0
##  [43] processx_3.5.3        goftest_1.2-2         tidygraph_1.2.0
##  [46] rlang_1.0.2           splines_4.1.1         lazyeval_0.2.2
##  [49] spatstat.geom_2.1-0   broom_0.7.6           yaml_2.3.5
##  [52] reshape2_1.4.4        abind_1.4-5           modelr_0.1.8
##  [55] backports_1.2.1       httpuv_1.6.1          tools_4.1.1
##  [58] ellipsis_0.3.2        spatstat.core_2.1-2   RColorBrewer_1.1-2
##  [61] ggridges_0.5.3        Rcpp_1.0.8            plyr_1.8.7
##  [64] ps_1.6.0              rpart_4.1-15          deldir_0.2-10
##  [67] pbapply_1.4-3         viridis_0.6.2         cowplot_1.1.1
##  [70] dynparam_1.0.2        zoo_1.8-9             haven_2.4.1
##  [73] ggrepel_0.9.1         cluster_2.1.2         fs_1.5.0
##  [76] magrittr_2.0.2        data.table_1.14.0     scattermore_0.7
##  [79] carrier_0.1.0         lmtest_0.9-38         reprex_2.0.1
```

```
##  [82] RANN_2.6.1               fitdistrplus_1.1-5    matrixStats_0.61.0
##  [85] hms_1.1.0                patchwork_1.1.1       mime_0.10
##  [88] evaluate_0.14            xtable_1.8-4          readxl_1.3.1
##  [91] gridExtra_2.3            testthat_3.0.2        compiler_4.1.1
##  [94] KernSmooth_2.23-20       crayon_1.5.1          htmltools_0.5.2
##  [97] proxyC_0.2.4             mgcv_1.8-36           later_1.2.0
## [100] RcppParallel_5.1.5       lubridate_1.7.10      DBI_1.1.1
## [103] tweenr_1.0.2             dbplyr_2.1.1          MASS_7.3-54
## [106] Matrix_1.3-4             cli_3.2.0             parallel_4.1.1
## [109] igraph_1.2.6             pkgconfig_2.0.3       plotly_4.9.4
## [112] spatstat.sparse_2.0-0 xml2_1.3.2            foreach_1.5.1
## [115] vipor_0.4.5              dynutils_1.0.9        rvest_1.0.2
## [118] digest_0.6.29            dyndimred_1.0.4       sctransform_0.3.2
## [121] RcppAnnoy_0.0.18         spatstat.data_2.1-0   rmarkdown_2.8
## [124] cellranger_1.1.0         leiden_0.3.8          uwot_0.1.10
## [127] shiny_1.7.1              lifecycle_1.0.0       nlme_3.1-152
## [130] jsonlite_1.8.0           desc_1.3.0            viridisLite_0.4.0
## [133] fansi_1.0.3              pillar_1.6.1          lattice_0.20-44
## [136] fastmap_1.1.0            httr_1.4.2            survival_3.2-11
## [139] waldo_0.2.5              glue_1.6.2            remotes_2.4.2
## [142] png_0.1-7                iterators_1.0.13      bit_4.0.4
## [145] ggforce_0.3.3            stringi_1.7.6         irlba_2.3.3
## [148] future.apply_1.7.0
```

References

K, Street, Risso D, Fletcher R, Das D, Ngai J, Yosef N, Purdom E, and Dudoit S. 2018. "Slingshot: Cell Lineage and Pseudotime Inference for Single-Cell Transcriptomics." *BMC Genomics* 19: 477.

W, Saelens, Cannoodt R, Todorov H, and Saeys Y. 2019. "A Comparison of Single-Cell Trajectory Inference Methods." *Nature Biotechnology* 37 (5): 547–54.
