# Supplementary material for: FGF8-mediated gene regulation affects regional identity in human cerebral organoids
Source: eLife. 2024 Nov 1;13:e98096. doi: 10.7554/eLife.98096 (PMC11581432; doi:10.7554/eLife.98096)
Supplement: Figure 6—figure supplement 1—source data 1. — Detailed report of enrichment analysis comparing different cell clusters in WNTi and WNTi + FGF8 organoids. [file elife-98096-fig6-figsupp1-data1.pdf]

S21306 – Enrichment analysis

Matthieu Jung

January 27, 2023

Enrichment analysis

Enrichment analyses of interest were performed using differentially expressed genes (DE) comparisons, the enrichment method and the terms indicated in Table 1. The analysis was performed using the Bioconductor package clusterProfiler version 4.2.0. Gene Set Enrichment Analysis (GSEA) method was proposed by Subramanian et al. (2005). For GSEA, all the genes included in the DE analysis and with a p-value adjusted were ranked by their log2 Fold-Change.

Table 1: **Enrichment analyses performed.**

Name of analysis	Name of comparison	Enrichment method	Term of interest	Description of term	Source of term
FGF8vsCTL_cls1-4 (GO:biological_process)	FGF8vsCTL_cls1-4	GSEA	GO:biological_process	Gene Ontology Biological Process (Ashburner et al. 2000)	Ensembl v98
FGF8vsCTL_cls1-4 (GO: cellular_component)	FGF8vsCTL_cls1-4	GSEA	GO:cellular_component	Gene Ontology Cellular Component (Ashburner et al. 2000)	Ensembl v98
FGF8vsCTL_cls1-4 (GO: molecular_function)	FGF8vsCTL_cls1-4	GSEA	GO: molecular_function	Gene Ontology Molecular Function (Ashburner et al. 2000)	Ensembl v98
FGF8vsCTL_cls2-5 (GO:biological_process)	FGF8vsCTL_cls2-5	GSEA	GO:biological_process	Gene Ontology Biological Process (Ashburner et al. 2000)	Ensembl v98
FGF8vsCTL_cls2-5 (GO: cellular_component)	FGF8vsCTL_cls2-5	GSEA	GO:cellular_component	Gene Ontology Cellular Component (Ashburner et al. 2000)	Ensembl v98
FGF8vsCTL_cls2-5 (GO: molecular_function)	FGF8vsCTL_cls2-5	GSEA	GO: molecular_function	Gene Ontology Molecular Function (Ashburner et al. 2000)	Ensembl v98
FGF8vsCTL_cls3 (GO:biological_process)	FGF8vsCTL_cls3	GSEA	GO:biological_process	Gene Ontology Biological Process (Ashburner et al. 2000)	Ensembl v98
FGF8vsCTL_cls3 (GO: cellular_component)	FGF8vsCTL_cls3	GSEA	GO:cellular_component	Gene Ontology Cellular Component (Ashburner et al. 2000)	Ensembl v98
FGF8vsCTL_cls3 (GO: molecular_function)	FGF8vsCTL_cls3	GSEA	GO: molecular_function	Gene Ontology Molecular Function (Ashburner et al. 2000)	Ensembl v98

Name of analysis	Name of comparison	Enrichment method	Term of interest	Description of term	Source of term
FGF8vsCTL_cls6-7 (GO:biological_process)	FGF8vsCTL_cls6-7	GSEA	GO:biological_process	Gene Ontology Biological Process (Ashburner et al. 2000)	Ensembl v98
FGF8vsCTL_cls6-7 (GO: cellular_component)	FGF8vsCTL_cls6-7	GSEA	GO:cellular_component	Gene Ontology Cellular Component (Ashburner et al. 2000)	Ensembl v98
FGF8vsCTL_cls6-7 (GO: molecular_function)	FGF8vsCTL_cls6-7	GSEA	GO: molecular_function	Gene Ontology Molecular Function (Ashburner et al. 2000)	Ensembl v98
FGF8vsCTL_cls12-15 (GO:biological_process)	FGF8vsCTL_cls12-15	GSEA	GO:biological_process	Gene Ontology Biological Process (Ashburner et al. 2000)	Ensembl v98
FGF8vsCTL_cls12-15 (GO: cellular_component)	FGF8vsCTL_cls12-15	GSEA	GO:cellular_component	Gene Ontology Cellular Component (Ashburner et al. 2000)	Ensembl v98
FGF8vsCTL_cls12-15 (GO: molecular_function)	FGF8vsCTL_cls12-15	GSEA	GO: molecular_function	Gene Ontology Molecular Function (Ashburner et al. 2000)	Ensembl v98
FGF8vsCTL_cls8-9 (GO:biological_process)	FGF8vsCTL_cls8-9	GSEA	GO:biological_process	Gene Ontology Biological Process (Ashburner et al. 2000)	Ensembl v98
FGF8vsCTL_cls8-9 (GO: cellular_component)	FGF8vsCTL_cls8-9	GSEA	GO:cellular_component	Gene Ontology Cellular Component (Ashburner et al. 2000)	Ensembl v98
FGF8vsCTL_cls8-9 (GO: molecular_function)	FGF8vsCTL_cls8-9	GSEA	GO: molecular_function	Gene Ontology Molecular Function (Ashburner et al. 2000)	Ensembl v98

Figure 1 to Figure 18 represent the results of these comparisons. Elements in italic are defined in *Files delivered* section. Enriched terms are ordered by their *adjusted p-value*, and if identical, by the absolute value of their *normalized enrichment score*.

Files delivered

For each enrichment analysis performed, a corresponding tsv file containing results of the analysis is available. Files contain the following columns:

Core enrichment Name of genes that contribute to the leading-edge subset within the gene set. This is the subset of genes that contributes most to the enrichment result.

Term description Description of the enriched term.

Enrichment score Degree to which a gene set is over-represented at the top or bottom of a ranked list of genes (Subramanian et al. 2005).

External link Web link to online information about the enriched term.

Term ID ID of the enriched term.

Normalized enrichment score Normalized enrichment score to account for the size of the set. (Subramanian et al. 2005)

Adjusted p-value P-value of the statistical test, adjusted for multiple testing.

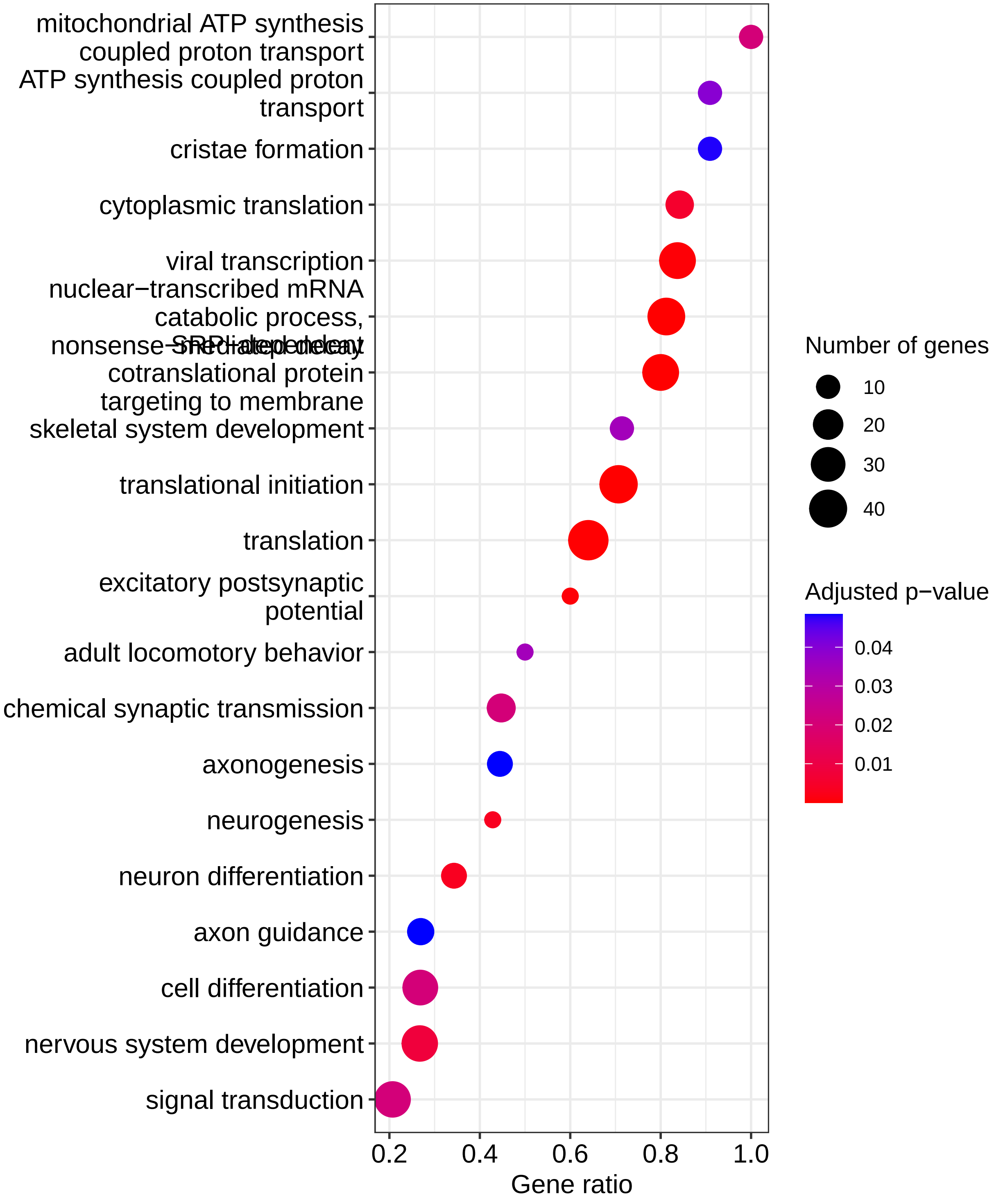

Figure 1: **FGF8vsCTL_cls1-4 (GO:biological_process) enrichment analysis.** Dotplot representing the *gene ratio* as a function of terms. Size of dot represents the *number of genes* annotated with that term, and color the *adjusted p-value* for that term. The top 20 terms are shown and are ordered on the y-axis by their *gene ratio*.

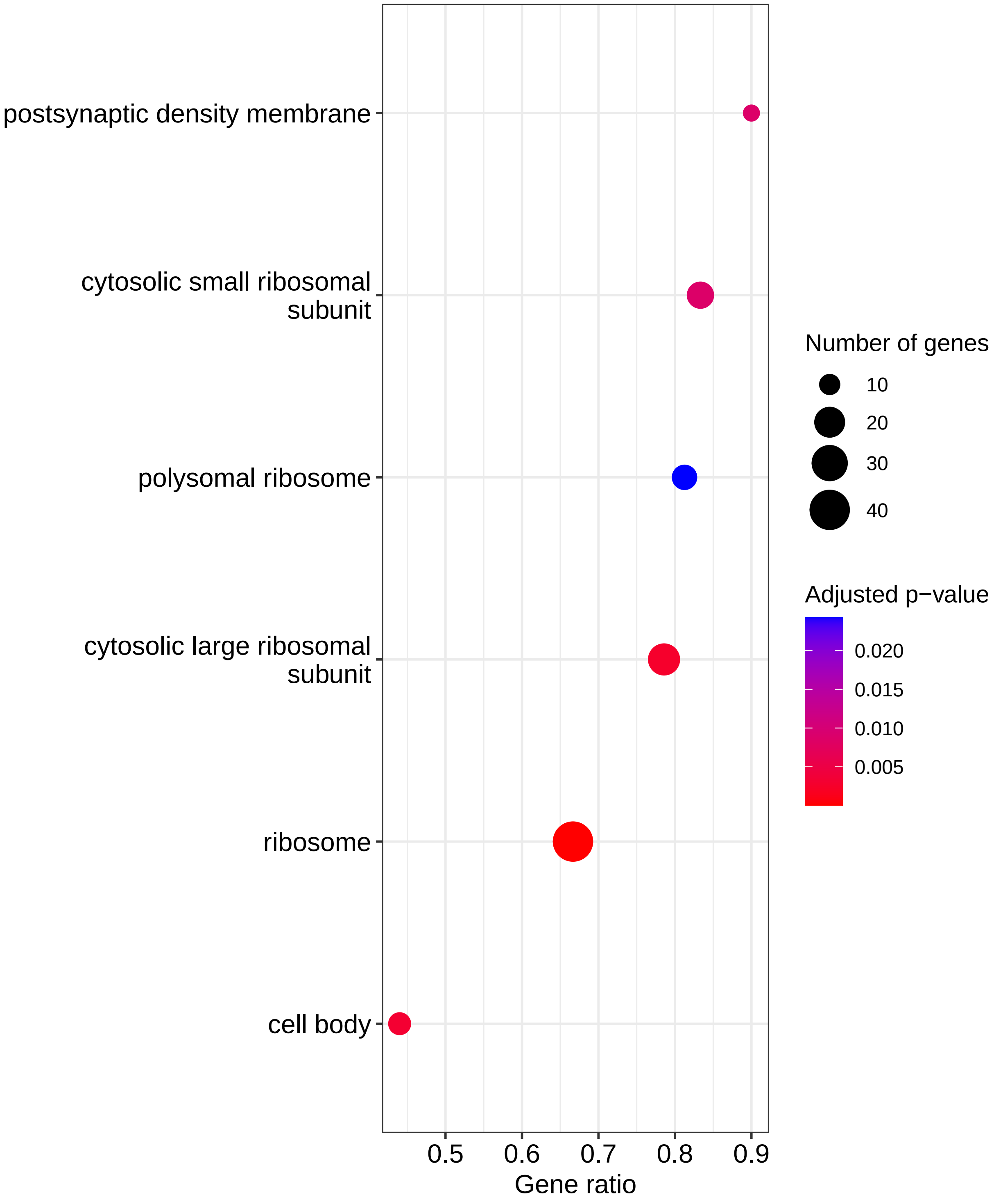

Figure 2: **FGF8vsCTL__cls1-4 (GO:cellular__component) enrichment analysis.** Dotplot representing the *gene ratio* as a function of terms. Size of dot represents the *number of genes* annotated with that term, and color the *adjusted p-value* for that term. The top 20 terms are shown and are ordered on the y-axis by their *gene ratio*.

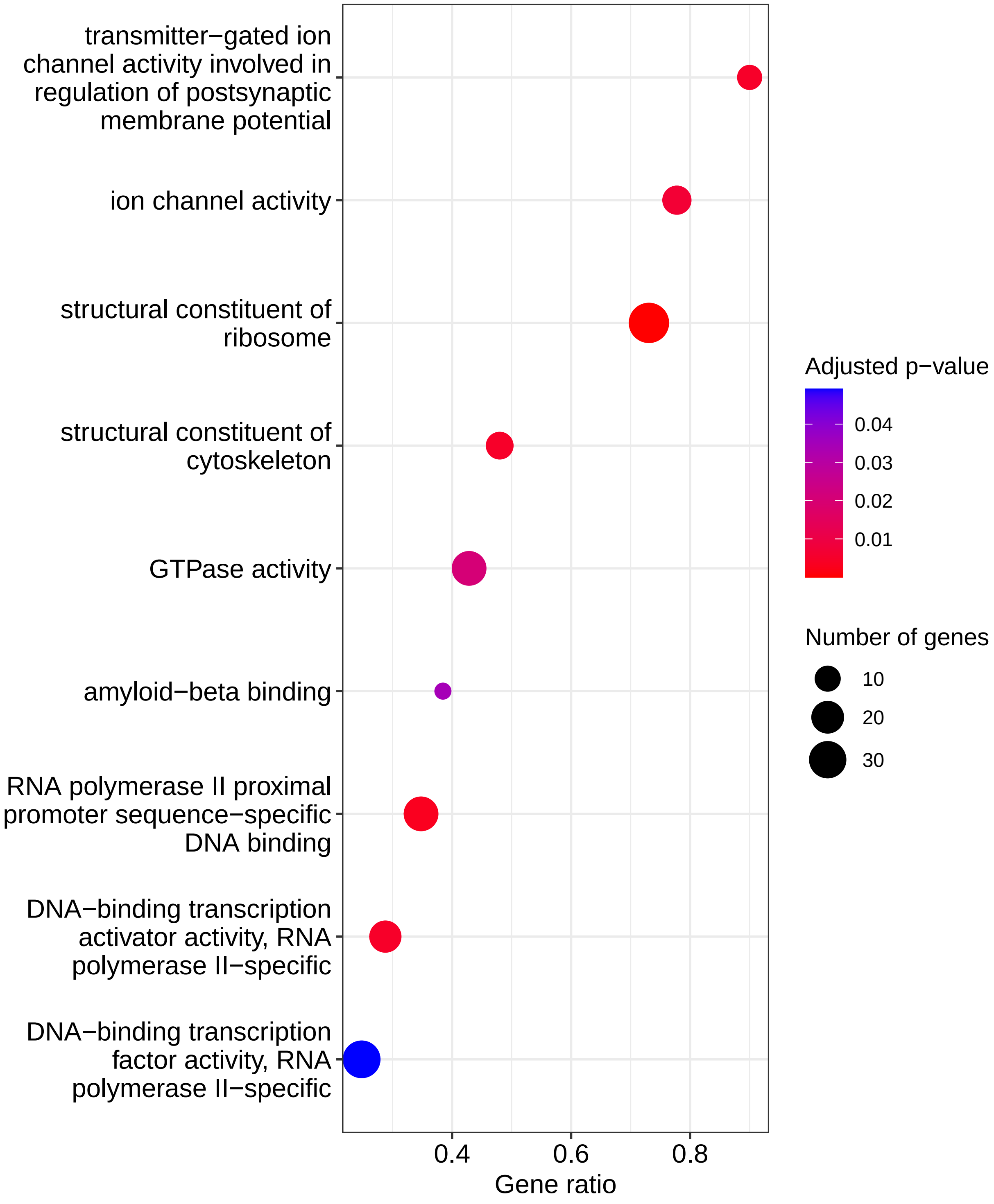

Figure 3: **FGF8vsCTL__cls1-4 (GO:molecular__function) enrichment analysis.** Dotplot representing the *gene ratio* as a function of terms. Size of dot represents the *number of genes* annotated with that term, and color the *adjusted p-value* for that term. The top 20 terms are shown and are ordered on the y-axis by their *gene ratio*.

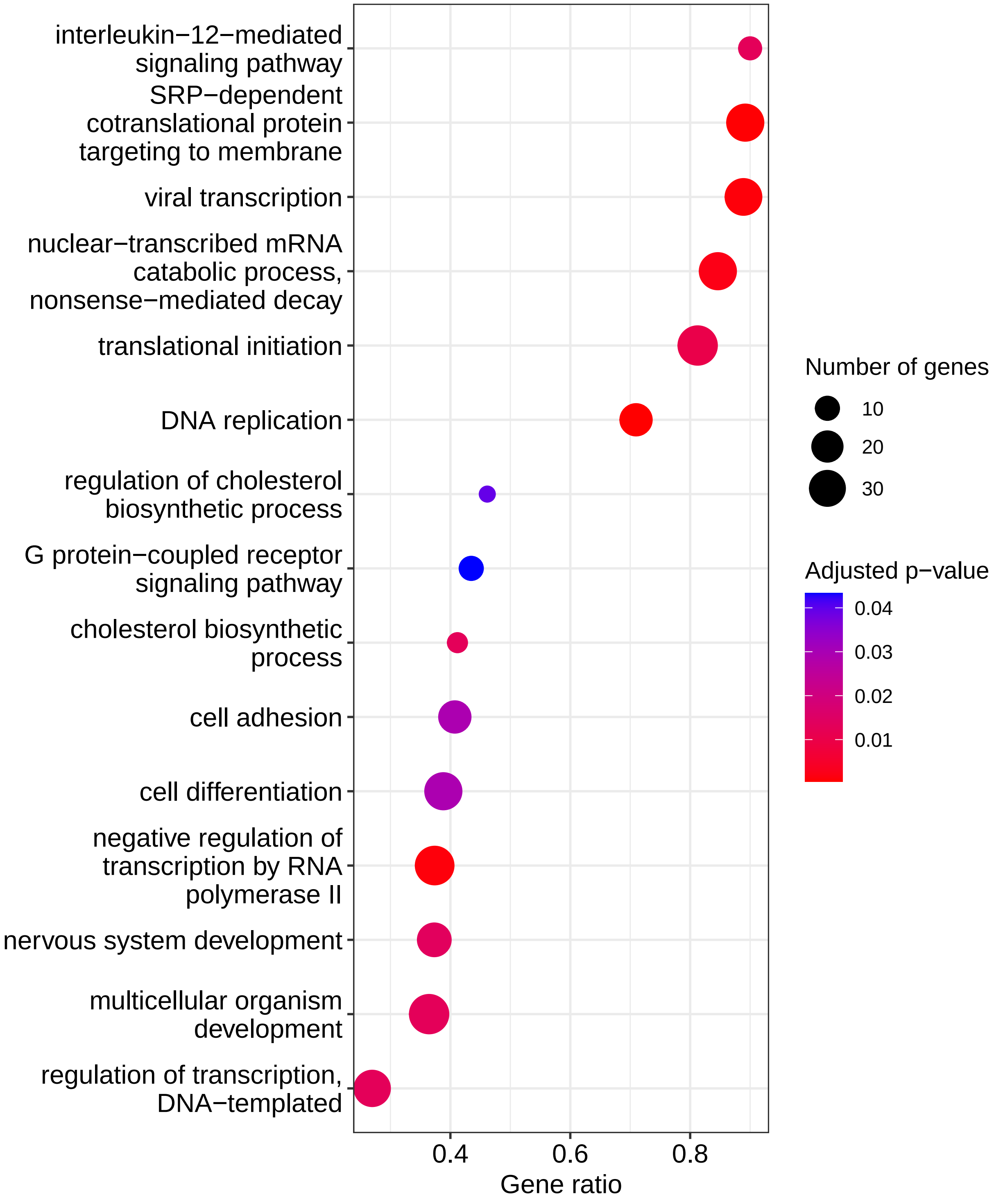

Figure 4: **FGF8vsCTL__cls2-5 (GO:biological__process) enrichment analysis.** Dotplot representing the *gene ratio* as a function of terms. Size of dot represents the *number of genes* annotated with that term, and color the *adjusted p-value* for that term. The top 20 terms are shown and are ordered on the y-axis by their *gene ratio*.

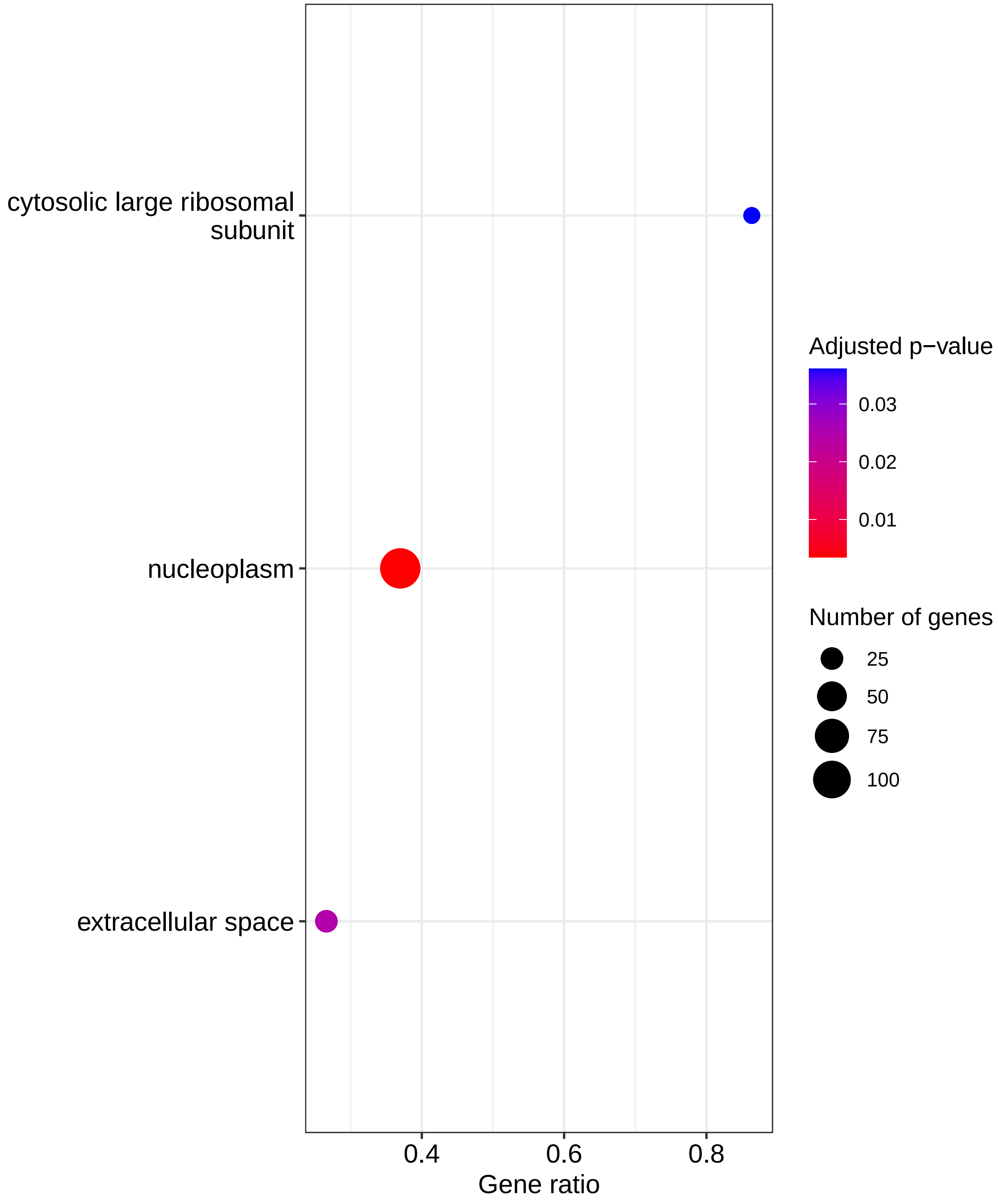

Figure 5: **FGF8vsCTL__cls2-5 (GO:cellular__component) enrichment analysis.** Dotplot representing the *gene ratio* as a function of terms. Size of dot represents the *number of genes* annotated with that term, and color the *adjusted p-value* for that term. The top 20 terms are shown and are ordered on the y-axis by their *gene ratio*.

No enrichment found.
Try changing analysis parameters.

Figure 6: **FGF8vsCTL_cls2-5 (GO:molecular_function) enrichment analysis.** Dotplot representing the *gene ratio* as a function of terms. Size of dot represents the *number of genes* annotated with that term, and color the *adjusted p-value* for that term. The top 20 terms are shown and are ordered on the y-axis by their *gene ratio*.

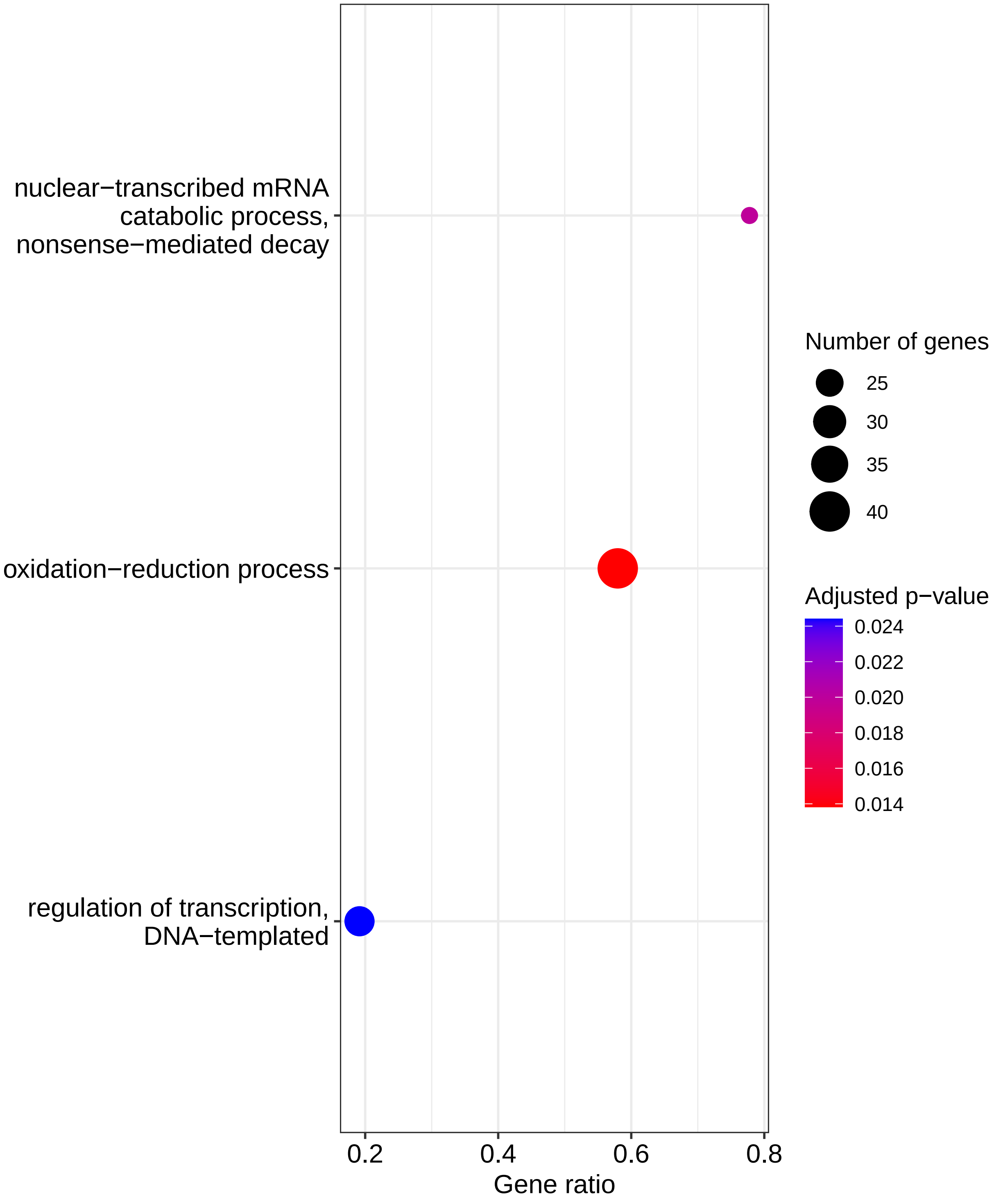

Figure 7: **FGF8vsCTL__cls3 (GO:biological__process) enrichment analysis.** Dotplot representing the *gene ratio* as a function of terms. Size of dot represents the *number of genes* annotated with that term, and color the *adjusted p-value* for that term. The top 20 terms are shown and are ordered on the y-axis by their *gene ratio*.

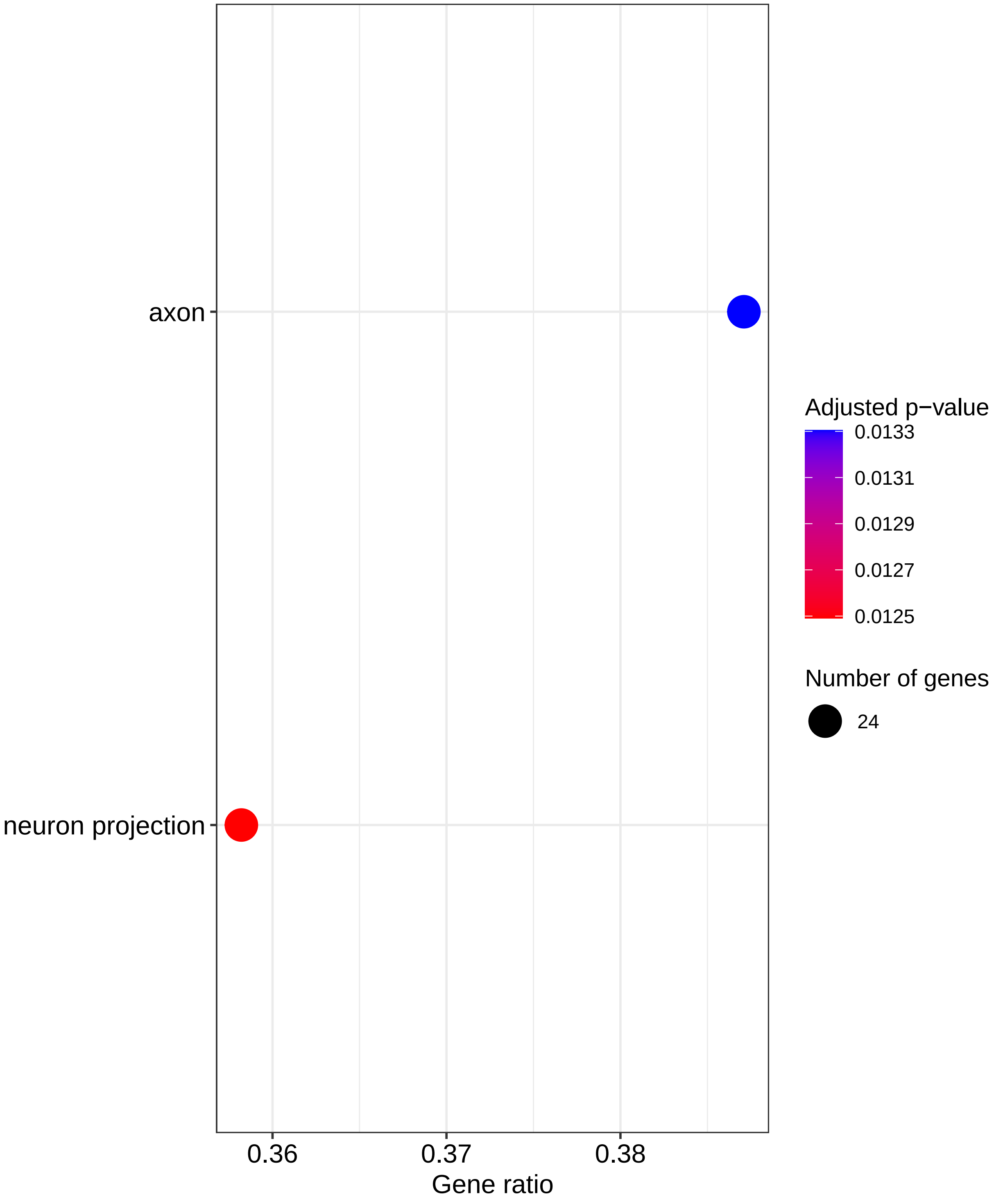

Figure 8: **FGF8vsCTL__cls3 (GO:cellular__component) enrichment analysis.** Dotplot representing the *gene ratio* as a function of terms. Size of dot represents the *number of genes* annotated with that term, and color the *adjusted p-value* for that term. The top 20 terms are shown and are ordered on the y-axis by their *gene ratio*.

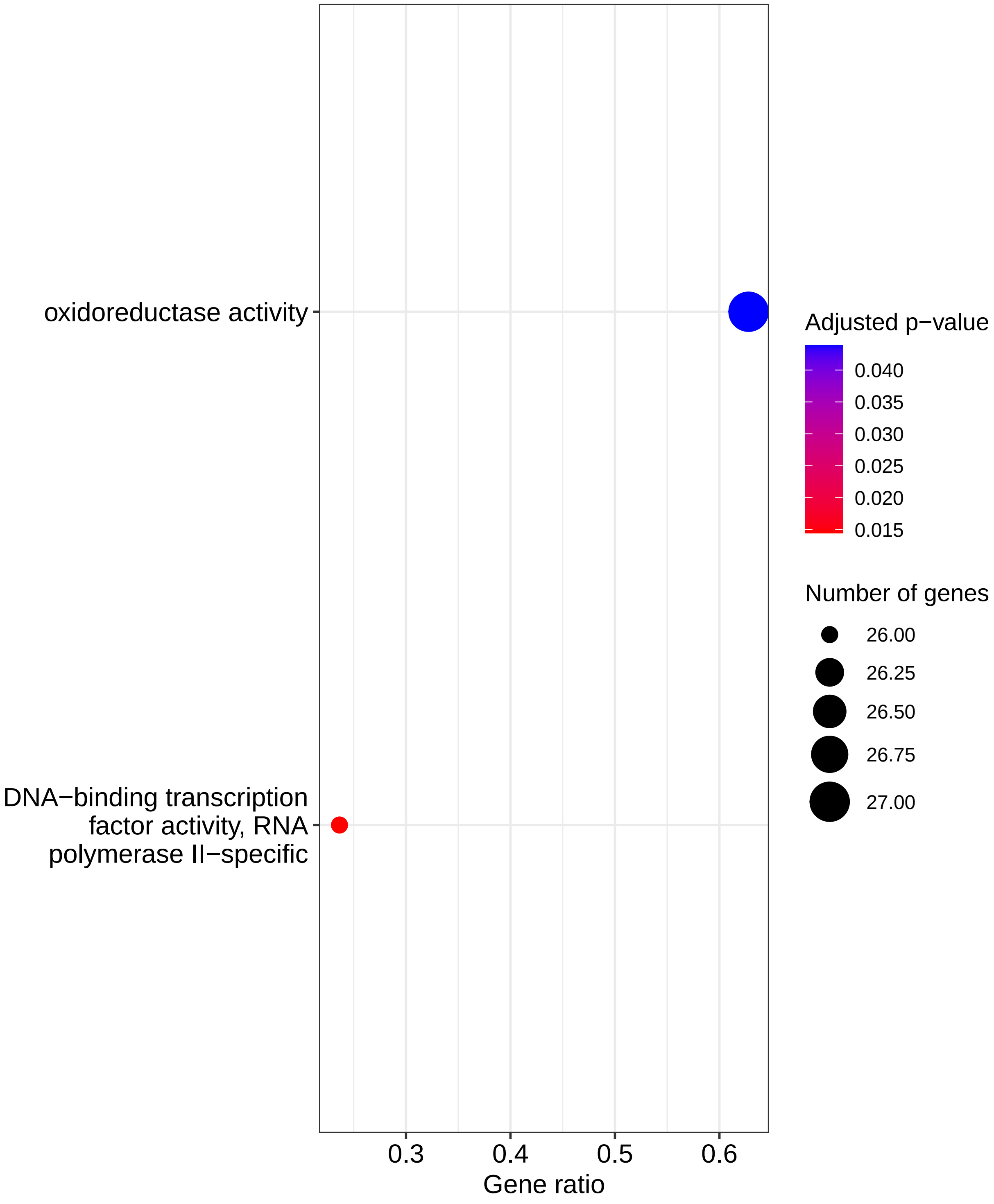

Figure 9: **FGF8vsCTL_cls3 (GO:molecular_function) enrichment analysis.** Dotplot representing the *gene ratio* as a function of terms. Size of dot represents the *number of genes* annotated with that term, and color the *adjusted p-value* for that term. The top 20 terms are shown and are ordered on the y-axis by their *gene ratio*.

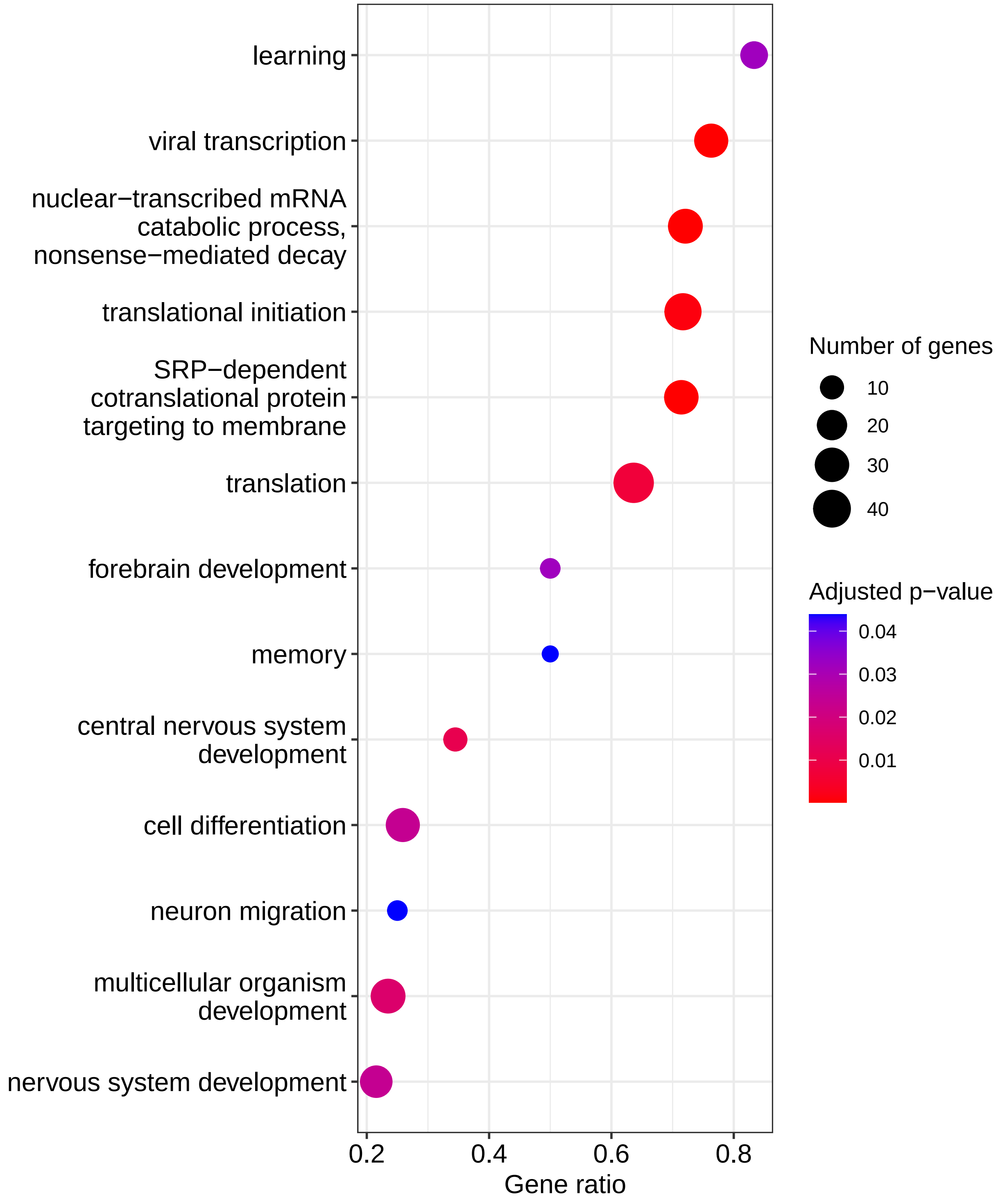

Figure 10: **FGF8vsCTL__cls6-7 (GO:biological__process) enrichment analysis.** Dotplot representing the *gene ratio* as a function of terms. Size of dot represents the *number of genes* annotated with that term, and color the *adjusted p-value* for that term. The top 20 terms are shown and are ordered on the y-axis by their *gene ratio*.

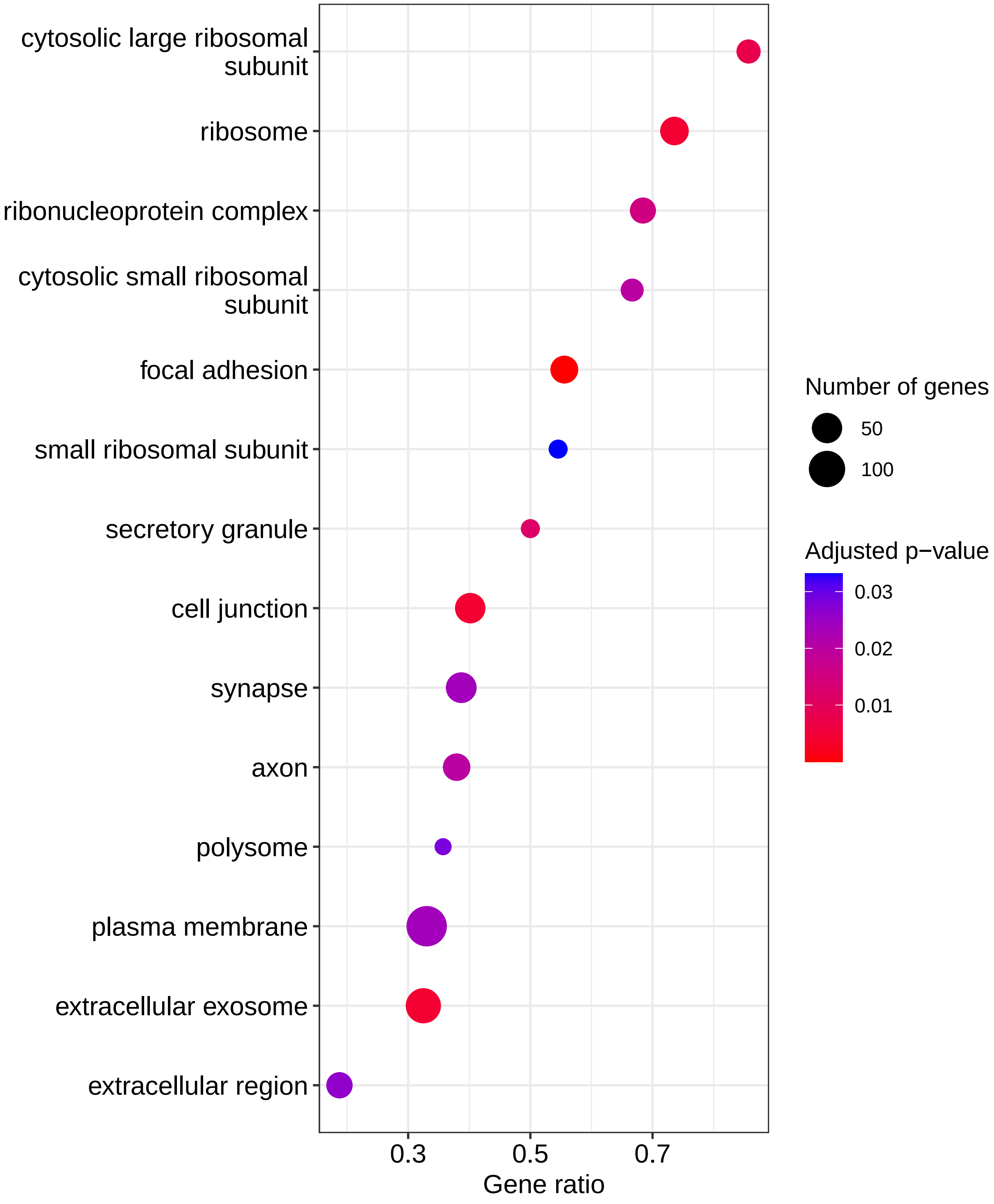

Figure 11: **FGF8vsCTL_cls6-7 (GO:cellular_component) enrichment analysis.** Dotplot representing the *gene ratio* as a function of terms. Size of dot represents the *number of genes* annotated with that term, and color the *adjusted p-value* for that term. The top 20 terms are shown and are ordered on the y-axis by their *gene ratio*.

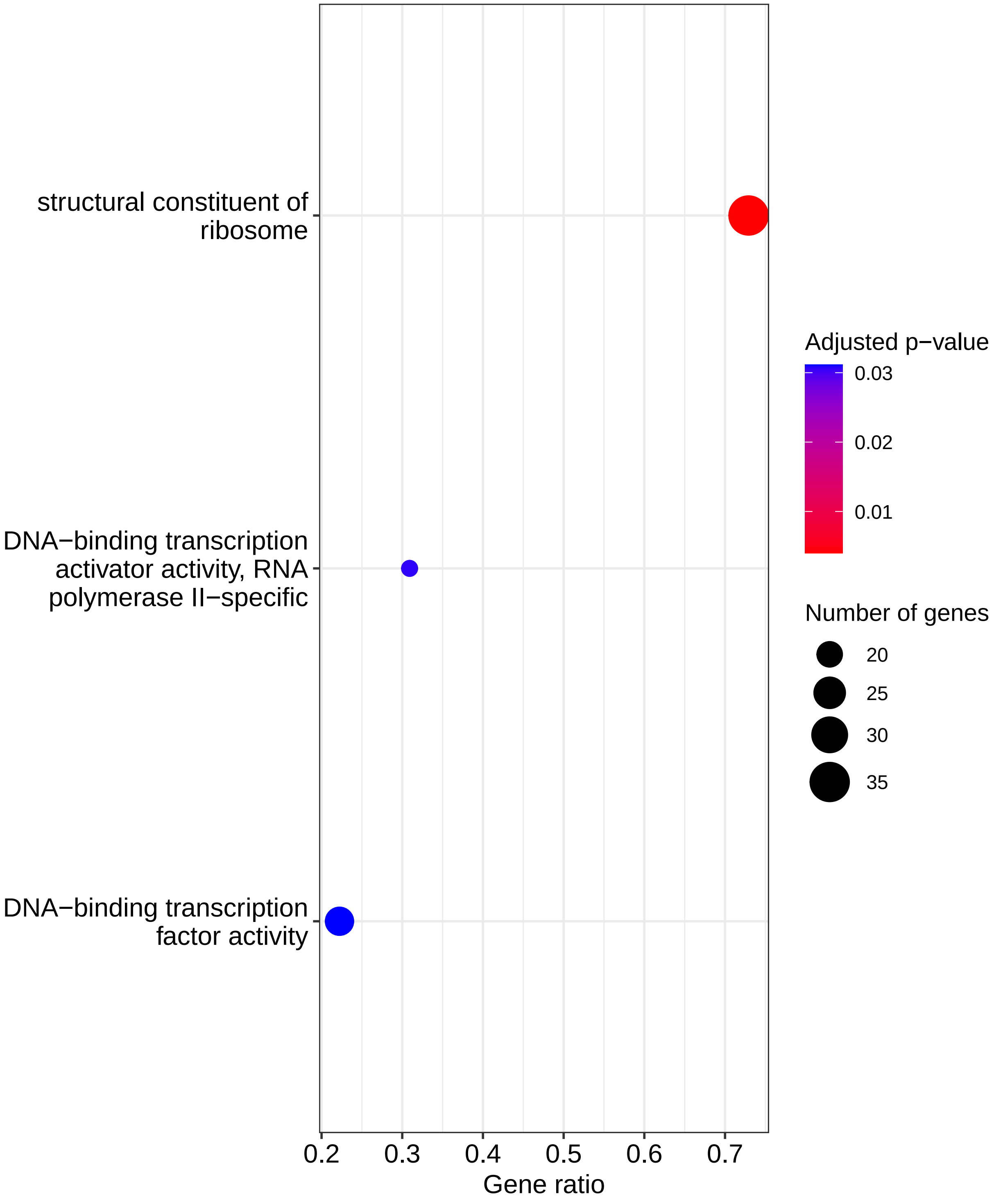

Figure 12: **FGF8vsCTL_cls6-7 (GO:molecular_function) enrichment analysis.** Dotplot representing the *gene ratio* as a function of terms. Size of dot represents the *number of genes* annotated with that term, and color the *adjusted p-value* for that term. The top 20 terms are shown and are ordered on the y-axis by their *gene ratio*.

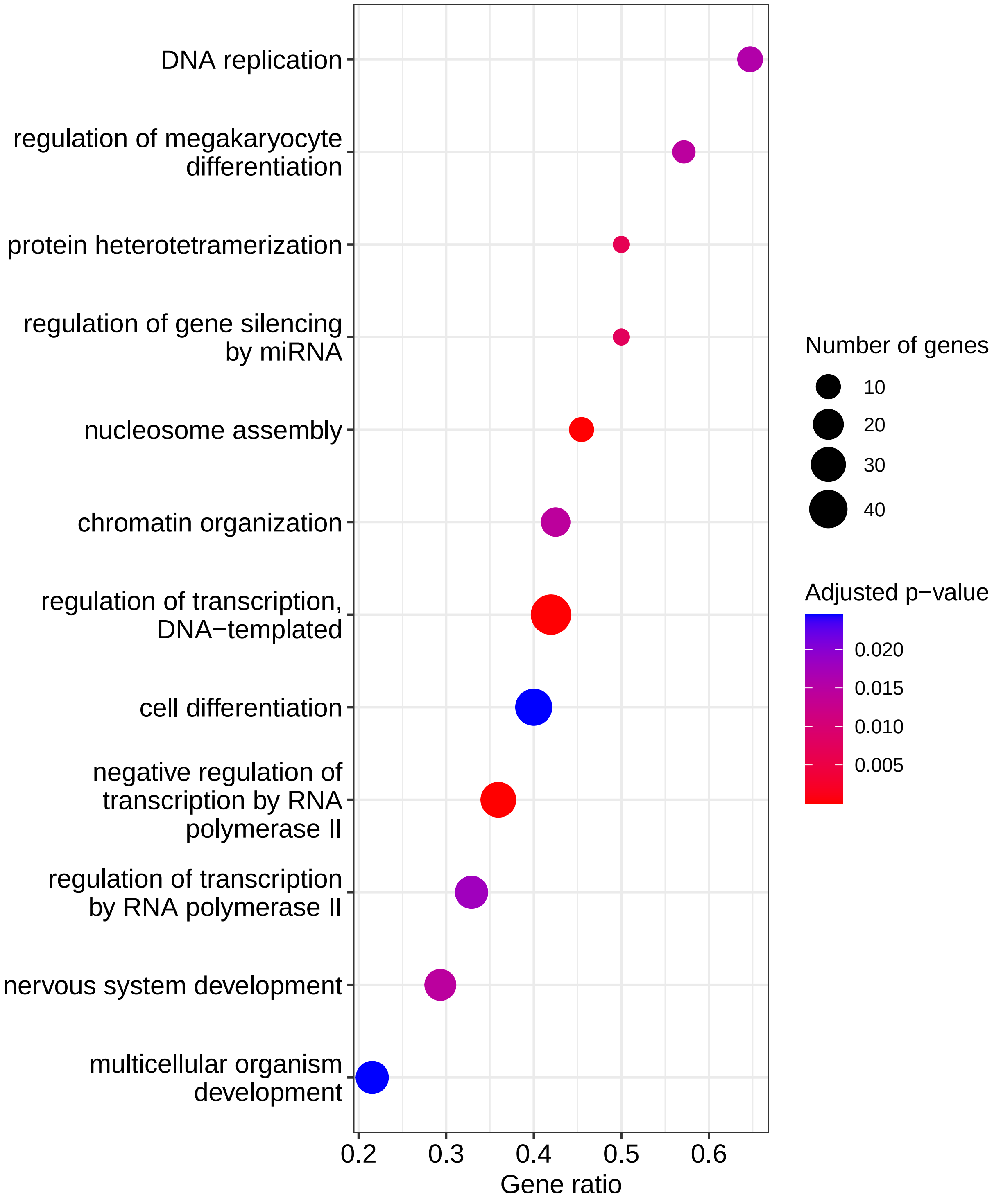

Figure 13: **FGF8vsCTL_cls12-15 (GO:biological_process) enrichment analysis.** Dotplot representing the *gene ratio* as a function of terms. Size of dot represents the *number of genes* annotated with that term, and color the *adjusted p-value* for that term. The top 20 terms are shown and are ordered on the y-axis by their *gene ratio*.

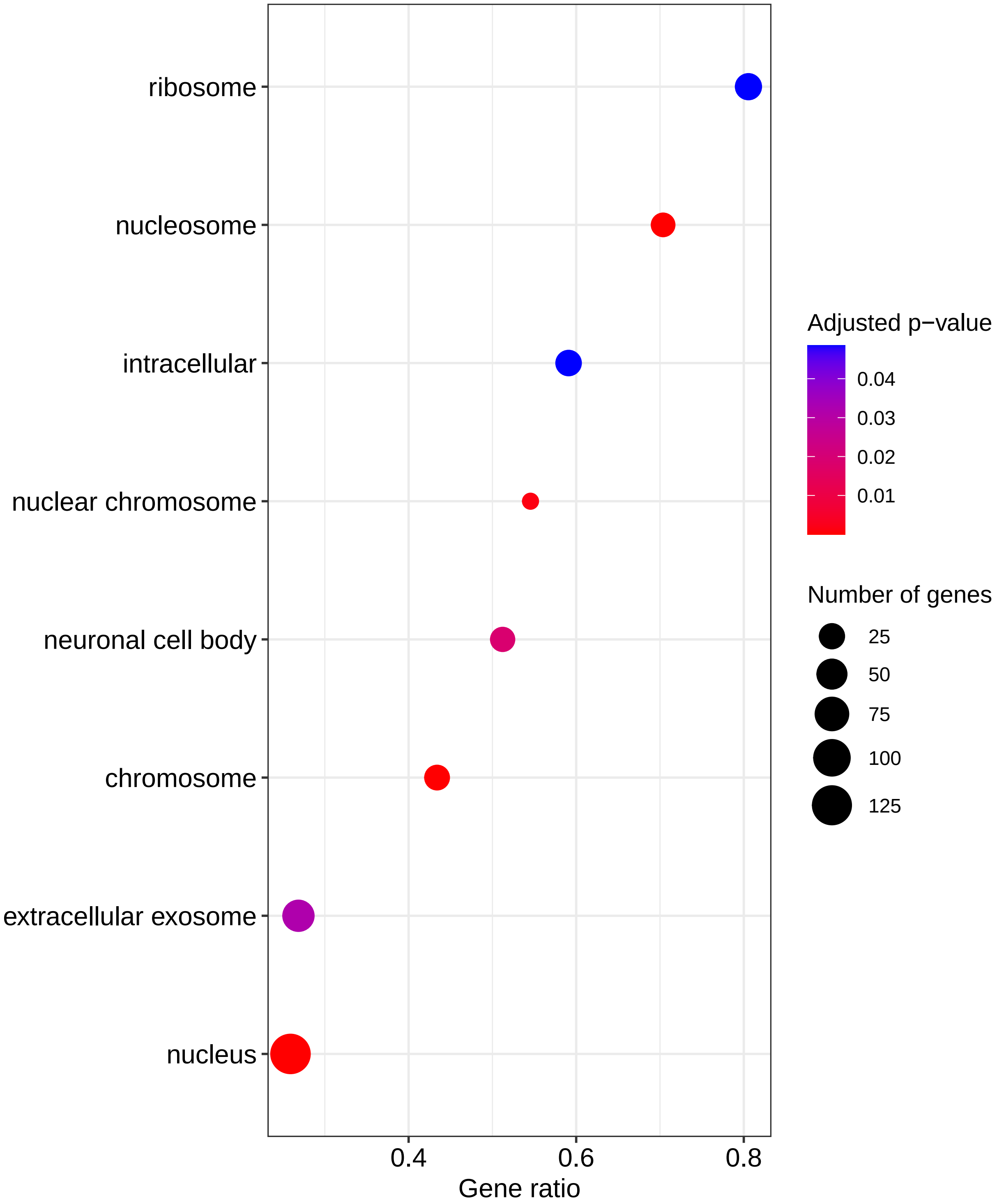

Figure 14: **FGF8vsCTL_cls12-15 (GO:cellular_component) enrichment analysis.** Dotplot representing the *gene ratio* as a function of terms. Size of dot represents the *number of genes* annotated with that term, and color the *adjusted p-value* for that term. The top 20 terms are shown and are ordered on the y-axis by their *gene ratio*.

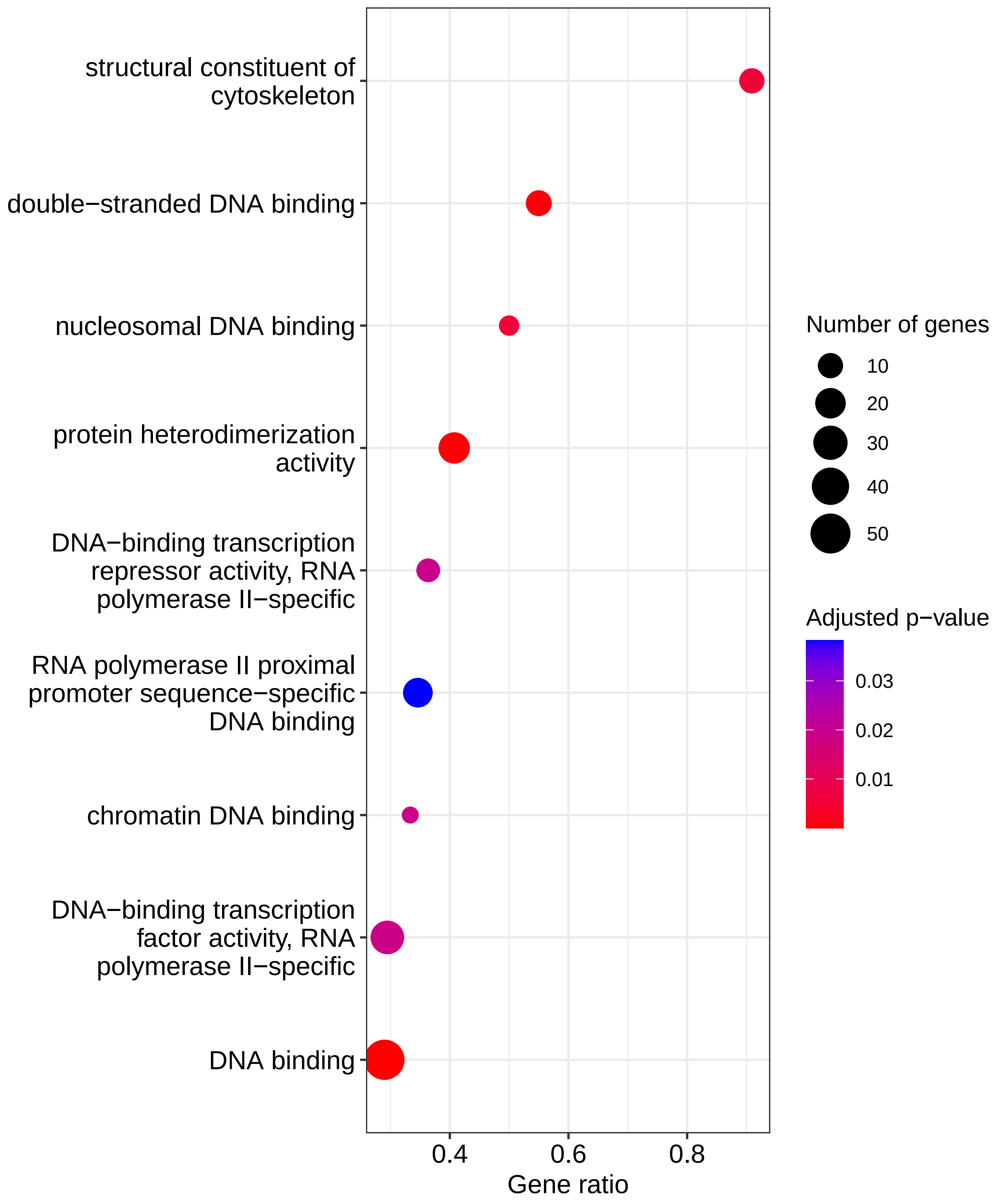

Figure 15: **FGF8vsCTL__cls12-15 (GO:molecular__function) enrichment analysis.** Dotplot representing the *gene ratio* as a function of terms. Size of dot represents the *number of genes* annotated with that term, and color the *adjusted p-value* for that term. The top 20 terms are shown and are ordered on the y-axis by their *gene ratio*.

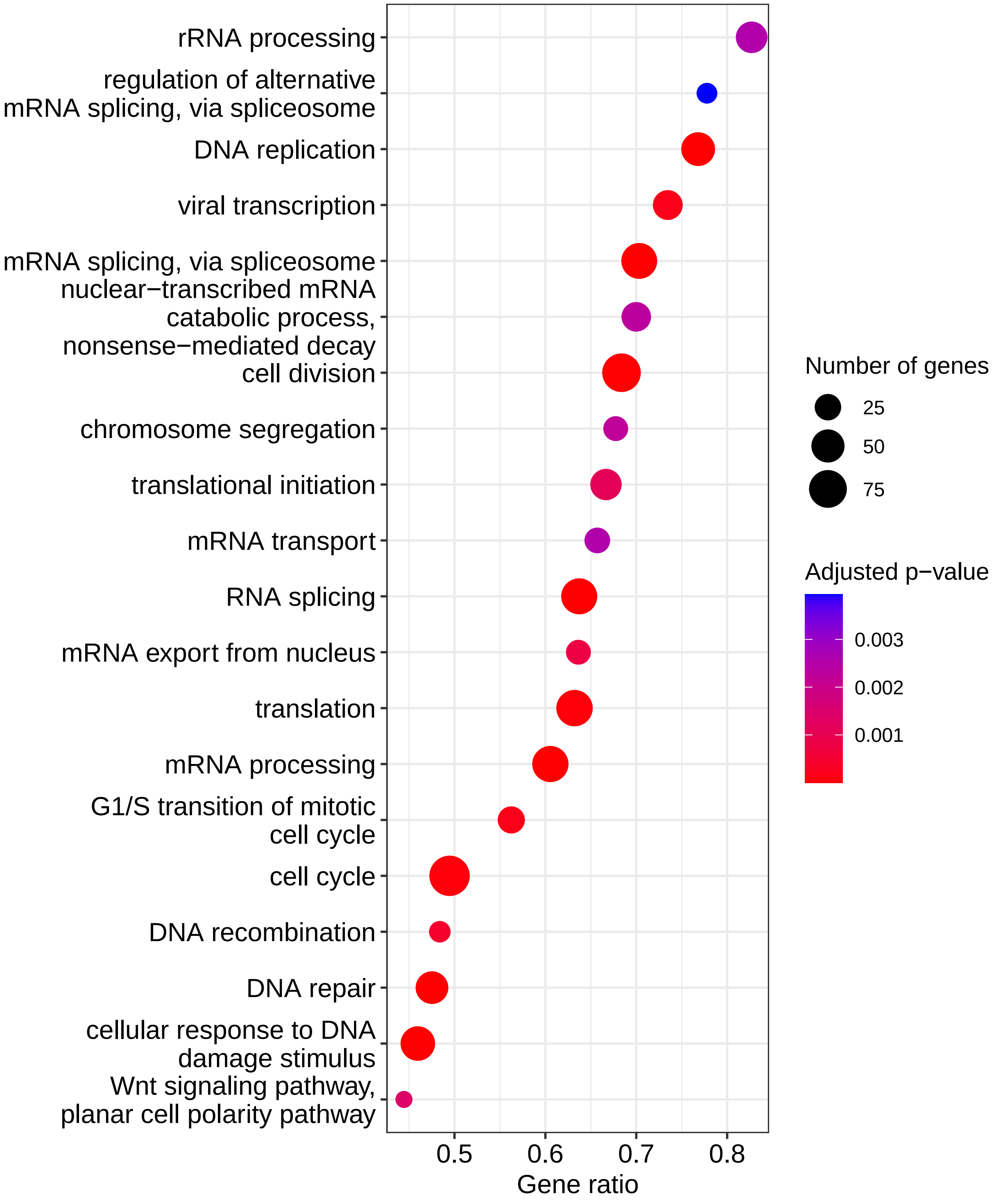

Figure 16: **FGF8vsCTL__cls8-9 (GO:biological__process) enrichment analysis.** Dotplot representing the *gene ratio* as a function of terms. Size of dot represents the *number of genes* annotated with that term, and color the *adjusted p-value* for that term. The top 20 terms are shown and are ordered on the y-axis by their *gene ratio*.

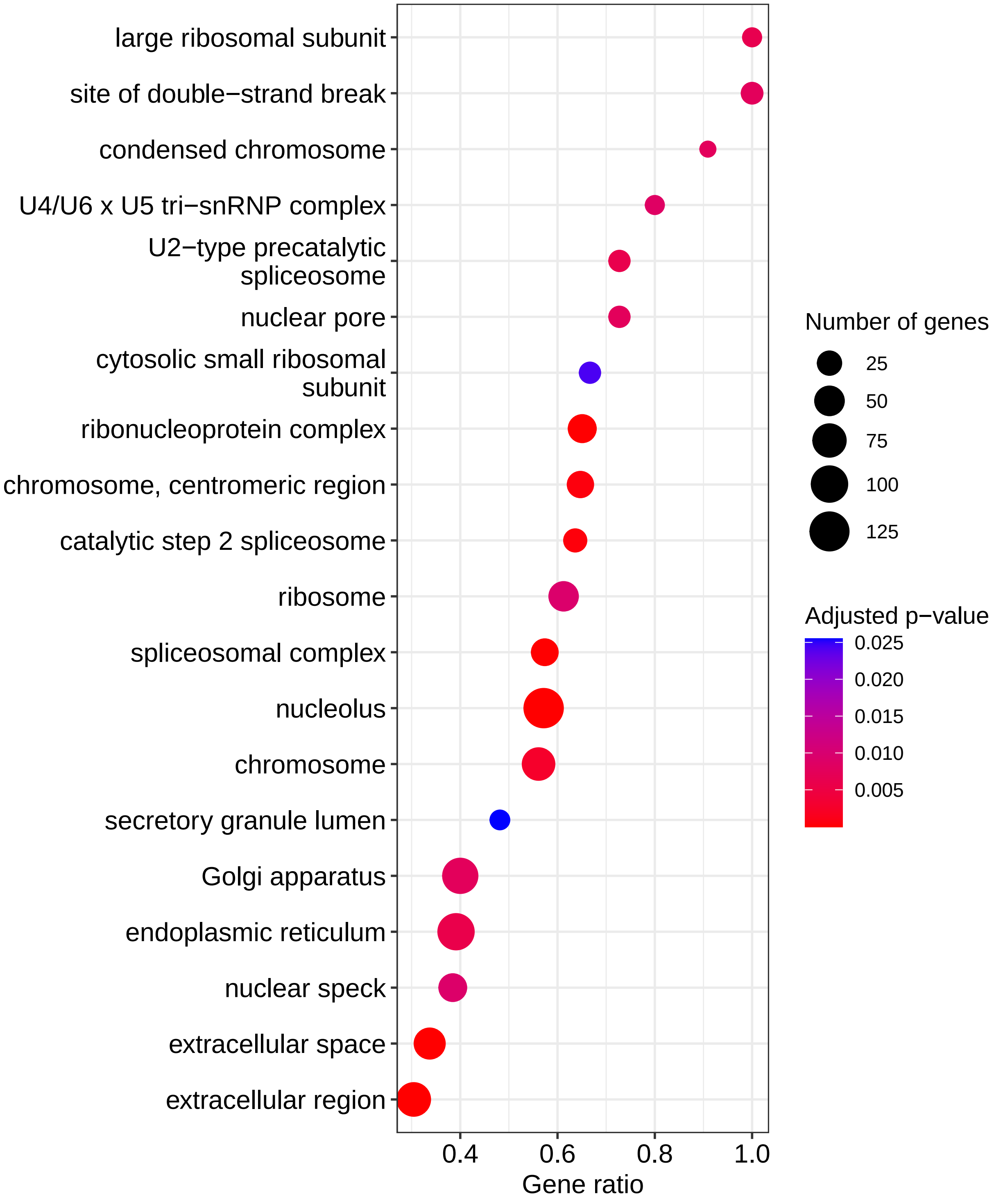

Figure 17: **FGF8vsCTL_cls8-9 (GO:cellular_component) enrichment analysis.** Dotplot representing the *gene ratio* as a function of terms. Size of dot represents the *number of genes* annotated with that term, and color the *adjusted p-value* for that term. The top 20 terms are shown and are ordered on the y-axis by their *gene ratio*.

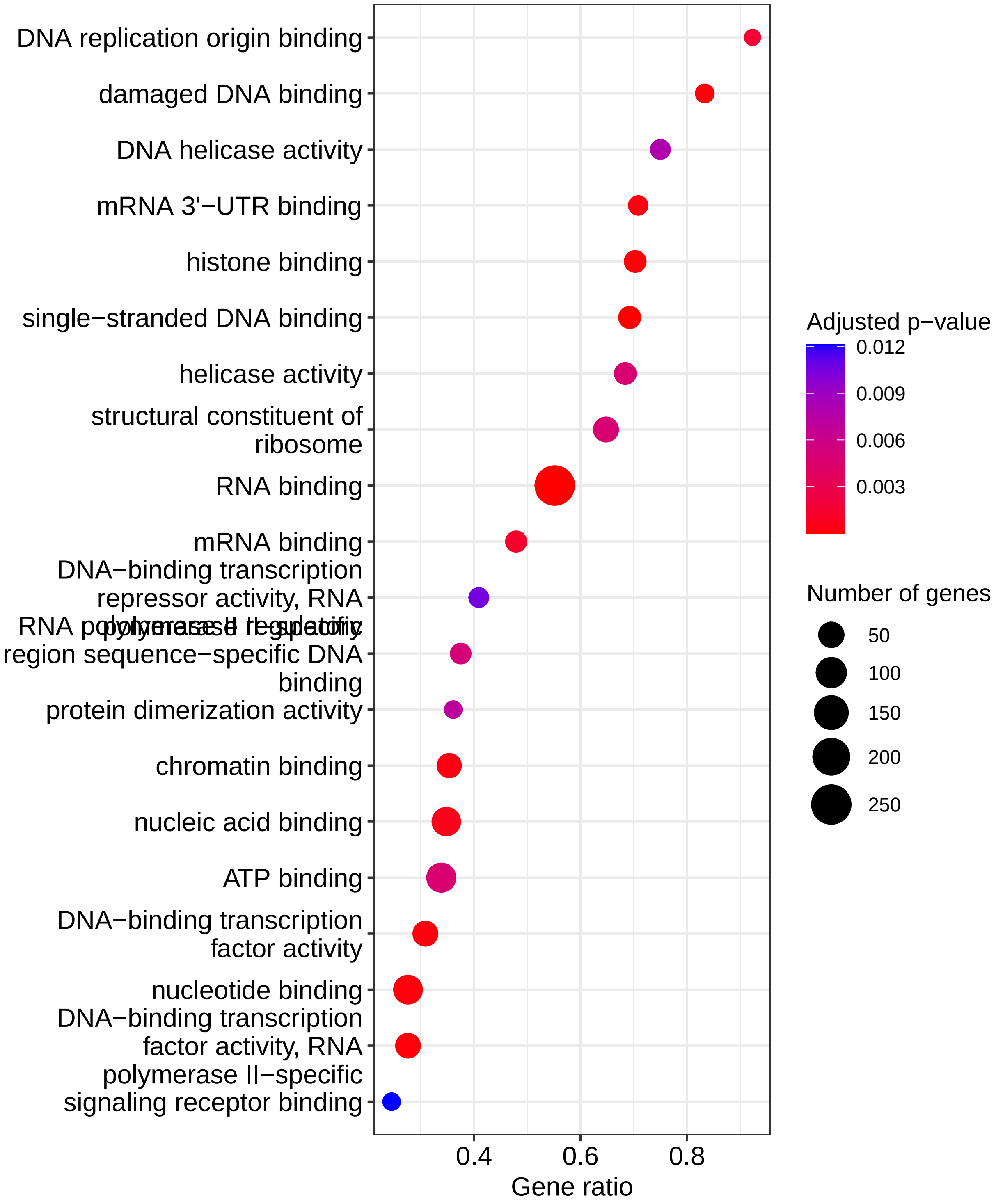

Figure 18: **FGF8vsCTL__cls8-9 (GO:molecular__function) enrichment analysis.** Dotplot representing the *gene ratio* as a function of terms. Size of dot represents the *number of genes* annotated with that term, and color the *adjusted p-value* for that term. The top 20 terms are shown and are ordered on the y-axis by their *gene ratio*.

P-value P-value of the statistical test.

Rank Position of maximum running enrichment score if enrichment score ≥ 0, or if enrichment score < 0, position (counting backwards) of minimum Running Enrichment Score.

Size of gene set Number of genes in the gene set.

R version package

```
## R version 4.1.3 (2022-03-10)
## Platform: x86_64-conda-linux-gnu (64-bit)
## Running under: Debian GNU/Linux 10 (buster)
##
## Matrix products: default
## BLAS/LAPACK: /shared/ngs/illumina/gilbartv/conda-envs/enrichment/lib/libopenblasp-r0.3.21.so
##
## locale:
##  [1] LC_CTYPE=fr_FR.UTF-8       LC_NUMERIC=C
##  [3] LC_TIME=fr_FR.UTF-8        LC_COLLATE=fr_FR.UTF-8
##  [5] LC_MONETARY=fr_FR.UTF-8    LC_MESSAGES=fr_FR.UTF-8
##  [7] LC_PAPER=fr_FR.UTF-8       LC_NAME=C
##  [9] LC_ADDRESS=C               LC_TELEPHONE=C
## [11] LC_MEASUREMENT=fr_FR.UTF-8 LC_IDENTIFICATION=C
##
## attached base packages:
## [1] grid      parallel  stats     graphics  grDevices utils     datasets
## [8] methods   base
##
## other attached packages:
##  [1] RColorBrewer_1.1-3    VennDiagram_1.6.20    futile.logger_1.4.3
##  [4] ggfortify_0.4.14      ggrepel_0.9.1         ggplot2_3.3.6
##  [7] xtable_1.8-4          readr_1.4.0           dplyr_1.0.10
## [10] stringr_1.4.1         msigdbr_7.5.1         ggridges_0.5.4
## [13] enrichplot_1.14.1     clusterProfiler_4.2.0 knitr_1.37
##
## loaded via a namespace (and not attached):
##   [1] fgsea_1.20.0           colorspace_2.0-3       ggtree_3.2.0
##   [4] ellipsis_0.3.2         qvalue_2.26.0          XVector_0.34.0
##   [7] aplot_0.1.8            farver_2.1.1           graphlayouts_0.8.2
##  [10] bit64_4.0.5            AnnotationDbi_1.56.1   fansi_1.0.3
##  [13] scatterpie_0.1.8       splines_4.1.3          cachem_1.0.6
##  [16] GOSemSim_2.20.0        polyclip_1.10-0        jsonlite_1.8.2
##  [19] GO.db_3.14.0           png_0.1-7              ggforce_0.3.3
##  [22] compiler_4.1.3         httr_1.4.4             assertthat_0.2.1
##  [25] Matrix_1.4-1           fastmap_1.1.0          lazyeval_0.2.2
##  [28] cli_3.4.1              formatR_1.12           tweenr_1.0.2
##  [31] htmltools_0.5.3        tools_4.1.3            igraph_1.2.6
##  [34] gtable_0.3.1           glue_1.6.2             GenomeInfoDbData_1.2.7
##  [37] reshape2_1.4.4         DO.db_2.9              fastmatch_1.1-0
##  [40] Rcpp_1.0.9             Biobase_2.54.0         vctrs_0.4.2
##  [43] Biostrings_2.62.0      ape_5.6-2              babelgene_22.9
##  [46] nlme_3.1-159           ggraph_2.1.0           xfun_0.30
##  [49] lifecycle_1.0.2        DOSE_3.20.0            zlibbioc_1.40.0
##  [52] MASS_7.3-58.1          scales_1.2.1           tidygraph_1.2.2
```

```
##  [55] hms_1.1.2             lambda.r_1.2.4         yaml_2.3.5
##  [58] memoise_2.0.1         gridExtra_2.3          downloader_0.4
##  [61] ggfun_0.0.7           yulab.utils_0.0.4      stringi_1.7.8
##  [64] RSQLite_2.2.8         highr_0.9              S4Vectors_0.32.4
##  [67] tidytree_0.4.1        BiocGenerics_0.40.0    BiocParallel_1.28.3
##  [70] GenomeInfoDb_1.30.0   rlang_1.0.6            pkgconfig_2.0.3
##  [73] bitops_1.0-7          evaluate_0.16          lattice_0.20-45
##  [76] purrr_0.3.5           labeling_0.4.2         treeio_1.18.0
##  [79] patchwork_1.1.1       shadowtext_0.1.2       bit_4.0.4
##  [82] tidyselect_1.1.2      plyr_1.8.7             magrittr_2.0.3
##  [85] R6_2.5.1              IRanges_2.28.0         generics_0.1.3
##  [88] DBI_1.1.3             pillar_1.8.1           withr_2.5.0
##  [91] KEGGREST_1.34.0       RCurl_1.98-1.9         tibble_3.1.8
##  [94] crayon_1.5.2          futile.options_1.0.1   utf8_1.2.2
##  [97] rmarkdown_2.8         viridis_0.6.2          data.table_1.14.2
## [100] blob_1.2.3            digest_0.6.29          tidyr_1.2.1
## [103] gridGraphics_0.5-1    stats4_4.1.3           munsell_0.5.0
## [106] viridisLite_0.4.1     ggplotify_0.1.0
```

References

Ashburner, Michael, Catherine A. Ball, Judith A. Blake, David Botstein, Heather Butler, J. Michael Cherry, Allan P. Davis, et al. 2000. "Gene Ontology: Tool for the Unification of Biology." *Nature Genetics* 25 (1): 25–29. https://doi.org/10.1038/75556.

Subramanian, Aravind, Pablo Tamayo, Vamsi K. Mootha, Sayan Mukherjee, Benjamin L. Ebert, Michael A. Gillette, Amanda Paulovich, et al. 2005. "Gene Set Enrichment Analysis: A Knowledge-Based Approach for Interpreting Genome-Wide Expression Profiles." *Proceedings of the National Academy of Sciences of the United States of America* 102 (43): 15545–50. https://doi.org/10.1073/pnas.0506580102.
