# Supplementary material for: FGF8-mediated gene regulation affects regional identity in human cerebral organoids
Source: eLife. 2024 Nov 1;13:e98096. doi: 10.7554/eLife.98096 (PMC11581432; doi:10.7554/eLife.98096)
Supplement: Figure 7—figure supplement 1—source data 1. — Detailed report on methods and data for annotating clusters/cells using SingleR, comparing in vitro organoids with in vivo fetal cortical regions, specifically assessing glutamatergic neuron (clusters 1, 3, 4) and progenitor (clusters 2, 5, 12, 14, 15) clusters. [file elife-98096-fig7-figsupp1-data1.pdf]

S21306 – Cluster/cell annotation using SingleR

Matthieu Jung

April 14, 2023

Single-cell dataset used in this report corresponds to the A02 dataset where only cells belonging to clusters 1, 2, 3, 4, 5, 12, 14 and 15 were kept. The reference dataset corresponds to primary cells dataset published by Speir et al. (2021) and where only cells belonging to GW18 age and not to hippocampus area were kept.

Both dataset were normalized using the `LogNormalize` method implemented in the Seurat R package version 4.3.0 (Hao et al. 2021). Then SingleR R package version 2.0.0 (Aran et al. 2019) was used in order to annotate Neurones_CTRL (clusters 1, 3 and 4 of CTRL cells), Neurones_FGF8 (clusters 1, 3 and 4 of FGF8 cells), Progeniteurs_CTRL (clusters 2, 5, 12, 14 and 15 of CTRL cells) and Progeniteurs_FGF8 (clusters 2, 5, 12, 14 and 15 of FGF8 cells) cells at the cluster/cell level with area annotation of reference dataset.

Figure 1 and 2 show a heatmap of the SingleR assignment scores as well as the corresponding inferred annotation for the clusters/cells. Scores allows users to inspect the confidence of the predicted labels across the dataset. Ideally, each cell/cluster (ie., column of the heatmap) should have one score that is obviously larger than the rest, indicating that it is unambiguously assigned to a single label. A spread of similar scores for a given cell/cluster indicates that the assignment is uncertain, though this may be acceptable if the uncertainty is distributed across similar cell/cluster types that cannot be easily resolved.

A CVS (comma-separated values) file `S21306_SingleR_cls.csv` is provided and contains the annotation inferred by SingleR in `SingleR_cell` and `SingleR_cluster` column. This file is intended to be opened with Loupe browser (10X Genomics), once the cloupe file is loaded.

R package version

```
## R version 4.2.2 (2022-10-31)
## Platform: x86_64-apple-darwin13.4.0 (64-bit)
## Running under: macOS Catalina 10.15.7
##
## Matrix products: default
## BLAS/LAPACK: /Users/jung/bin/miniconda3/envs/rstudio/lib/libopenblasp-r0.3.21.dylib
##
## locale:
## [1] fr_FR.UTF-8/fr_FR.UTF-8/fr_FR.UTF-8/C/fr_FR.UTF-8/fr_FR.UTF-8
##
## attached base packages:
## [1] stats4    stats     graphics  grDevices utils     datasets  methods
## [8] base
##
## other attached packages:
##  [1] SeuratObject_4.1.3          Seurat_4.3.0
##  [3] SingleR_2.0.0               SummarizedExperiment_1.28.0
##  [5] Biobase_2.58.0              GenomicRanges_1.50.0
##  [7] GenomeInfoDb_1.34.1         IRanges_2.32.0
##  [9] S4Vectors_0.36.0            BiocGenerics_0.44.0
```

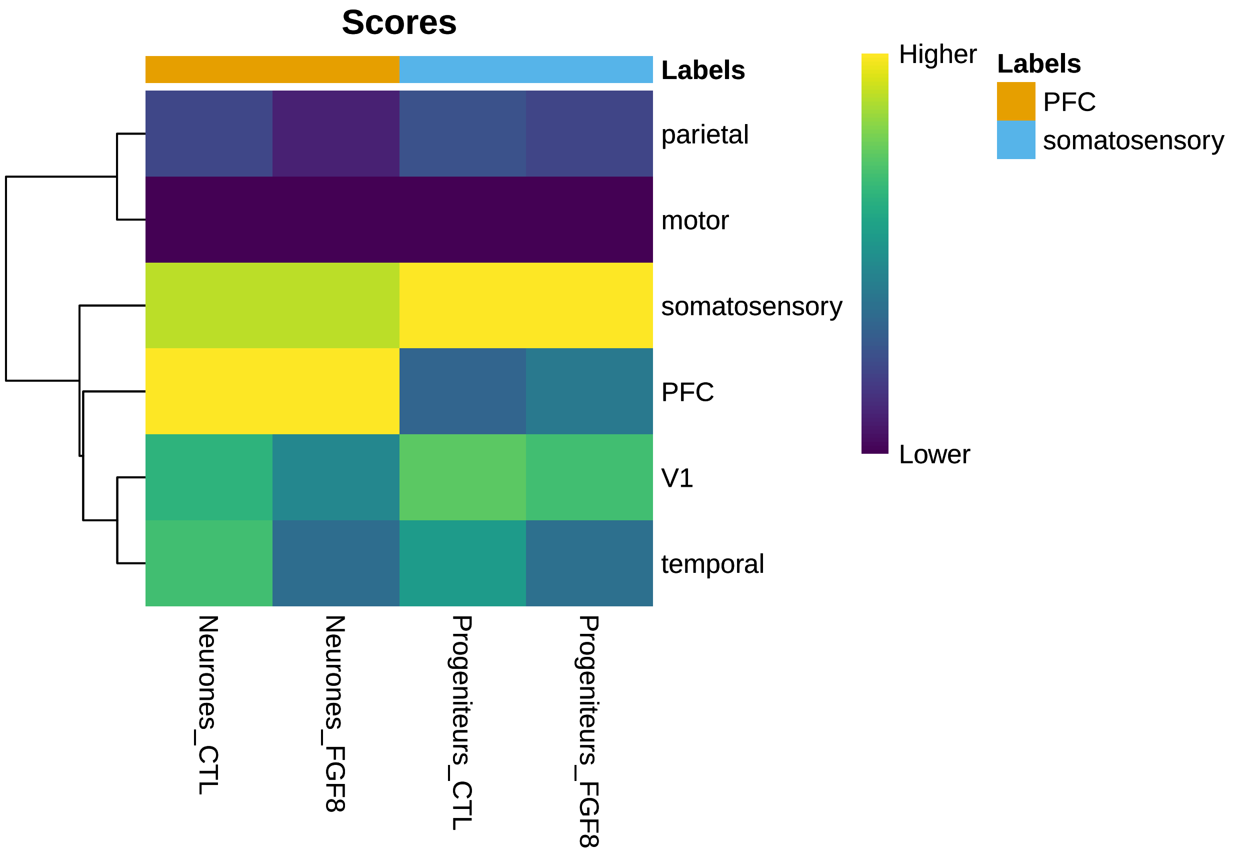

Figure 1: **Heatmap of the SingleR assignment scores for cluster level assignment.**

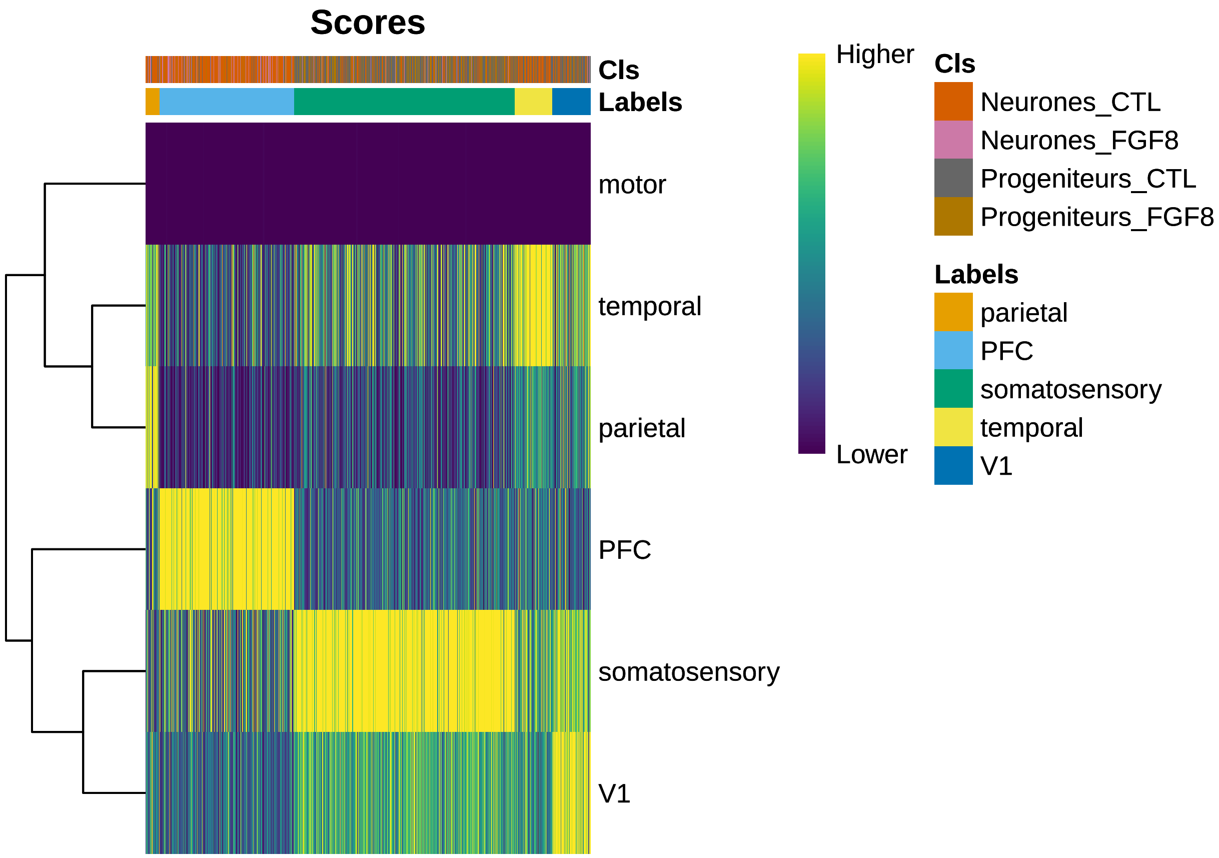

Figure 2: **Heatmap of the SingleR assignment scores for cell level assignment.**

```
## [11] MatrixGenerics_1.10.0       matrixStats_0.63.0
## [13] forcats_0.5.2               stringr_1.5.0
## [15] dplyr_1.0.10                purrr_0.3.5
## [17] readr_2.1.3                 tidyr_1.2.1
## [19] tibble_3.1.8                ggplot2_3.4.0
## [21] tidyverse_1.3.2
##
## loaded via a namespace (and not attached):
##   [1] utf8_1.2.2                 spatstat.explore_3.0-5
##   [3] reticulate_1.26            tidyselect_1.2.0
##   [5] htmlwidgets_1.5.4          grid_4.2.2
##   [7] BiocParallel_1.32.0        Rtsne_0.16
##   [9] munsell_0.5.0              ScaledMatrix_1.6.0
##  [11] codetools_0.2-18           ica_1.0-3
##  [13] statmod_1.4.37             scran_1.26.0
##  [15] future_1.29.0              miniUI_0.1.1.1
##  [17] withr_2.5.0                spatstat.random_3.0-1
##  [19] colorspace_2.0-3           progressr_0.11.0
##  [21] highr_0.9                  knitr_1.41
##  [23] rstudioapi_0.14            SingleCellExperiment_1.20.0
##  [25] ROCR_1.0-11                tensor_1.5
##  [27] listenv_0.8.0              GenomeInfoDbData_1.2.9
##  [29] polyclip_1.10-4            farver_2.1.1
##  [31] pheatmap_1.0.12            bit64_4.0.5
##  [33] parallelly_1.32.1          vctrs_0.5.1
##  [35] generics_0.1.3             xfun_0.35
##  [37] timechange_0.1.1           R6_2.5.1
##  [39] rsvd_1.0.5                 locfit_1.5-9.6
##  [41] bitops_1.0-7               spatstat.utils_3.0-1
##  [43] DelayedArray_0.24.0        assertthat_0.2.1
##  [45] promises_1.2.0.1           scales_1.2.1
##  [47] vroom_1.6.0                googlesheets4_1.0.1
##  [49] gtable_0.3.1               beachmat_2.14.0
##  [51] globals_0.16.2             goftest_1.2-3
##  [53] rlang_1.0.6                splines_4.2.2
##  [55] lazyeval_0.2.2             gargle_1.2.1
##  [57] spatstat.geom_3.0-3        broom_1.0.1
##  [59] yaml_2.3.6                 reshape2_1.4.4
##  [61] abind_1.4-5                modelr_0.1.10
##  [63] backports_1.4.1            httpuv_1.6.6
##  [65] tools_4.2.2                ellipsis_0.3.2
##  [67] RColorBrewer_1.1-3         ggridges_0.5.4
##  [69] Rcpp_1.0.9                 plyr_1.8.8
##  [71] sparseMatrixStats_1.10.0   zlibbioc_1.44.0
##  [73] RCurl_1.98-1.9             deldir_1.0-6
##  [75] viridis_0.6.2              pbapply_1.6-0
##  [77] cowplot_1.1.1              zoo_1.8-11
##  [79] haven_2.5.1                ggrepel_0.9.2
##  [81] cluster_2.1.4              fs_1.5.2
##  [83] magrittr_2.0.3             data.table_1.14.6
##  [85] scattermore_0.8            lmtest_0.9-40
##  [87] reprex_2.0.2               RANN_2.6.1
##  [89] googledrive_2.0.0          fitdistrplus_1.1-8
##  [91] hms_1.1.2                  patchwork_1.1.2
```

```
##  [93] mime_0.12                  evaluate_0.18
##  [95] xtable_1.8-4               readxl_1.4.1
##  [97] gridExtra_2.3              compiler_4.2.2
##  [99] KernSmooth_2.23-20         crayon_1.5.2
## [101] htmltools_0.5.4            later_1.3.0
## [103] tzdb_0.3.0                 lubridate_1.9.0
## [105] DBI_1.1.3                  dbplyr_2.2.1
## [107] MASS_7.3-58.1              Matrix_1.5-3
## [109] cli_3.4.1                  metapod_1.6.0
## [111] parallel_4.2.2             igraph_1.3.5
## [113] pkgconfig_2.0.3            sp_1.5-1
## [115] scuttle_1.8.0              plotly_4.10.1
## [117] spatstat.sparse_3.0-0      xml2_1.3.3
## [119] dqrng_0.3.0                XVector_0.38.0
## [121] rvest_1.0.3                digest_0.6.30
## [123] sctransform_0.3.5          RcppAnnoy_0.0.20
## [125] spatstat.data_3.0-0        rmarkdown_2.18
## [127] cellranger_1.1.0           leiden_0.4.3
## [129] uwot_0.1.14                edgeR_3.40.0
## [131] DelayedMatrixStats_1.20.0  shiny_1.7.3
## [133] lifecycle_1.0.3            nlme_3.1-160
## [135] jsonlite_1.8.4             BiocNeighbors_1.16.0
## [137] viridisLite_0.4.1          limma_3.54.0
## [139] fansi_1.0.3                pillar_1.8.1
## [141] lattice_0.20-45            fastmap_1.1.0
## [143] httr_1.4.4                 survival_3.4-0
## [145] glue_1.6.2                 png_0.1-8
## [147] bluster_1.8.0              bit_4.0.5
## [149] stringi_1.7.8              BiocSingular_1.14.0
## [151] irlba_2.3.5.1              future.apply_1.10.0
```

References

Aran, Dvir, Agnieszka P. Looney, Leqian Liu, Esther Wu, Valerie Fong, Austin Hsu, Suzanna Chak, et al. 2019. "Reference-Based Analysis of Lung Single-Cell Sequencing Reveals a Transitional Profibrotic Macrophage." *Nat. Immunol.* 20: 163–72. https://doi.org/10.1038/s41590-018-0276-y.

Hao, Yuhan, Stephanie Hao, Erica Andersen-Nissen, William M. Mauck III, Shiwei Zheng, Andrew Butler, Maddie J. Lee, et al. 2021. "Integrated Analysis of Multimodal Single-Cell Data." *Cell*. https://doi.org/10.1016/j.cell.2021.04.048.

Speir, Matthew L, Aparna Bhaduri, Nikolay S Markov, Pablo Moreno, Tomasz J Nowakowski, Irene Papatheodorou, Alex A Pollen, et al. 2021. "UCSC Cell Browser: visualize your single-cell data." *Bioinformatics* 37 (23): 4578–80. https://doi.org/10.1093/bioinformatics/btab503.
