# Supplementary material for: FGF8-mediated gene regulation affects regional identity in human cerebral organoids
Source: eLife. 2024 Nov 1;13:e98096. doi: 10.7554/eLife.98096 (PMC11581432; doi:10.7554/eLife.98096)
Supplement: MDAR checklist [file elife-98096-mdarchecklist1.pdf]

eLife

Materials Design Analysis Reporting (MDAR) Checklist for Authors

The MDAR framework establishes a minimum set of requirements in transparent reporting mainly applicable to studies in the life sciences.

eLife asks authors to **provide detailed information within their article** to facilitate the interpretation and replication of their work. Authors can also upload supporting materials to comply with relevant reporting guidelines for health-related research (see EQUATOR Network), life science research (see the BioSharing Information Resource), or animal research (see the ARRIVE Guidelines and the STRANGE Framework; for details, see *eLife*'s Journal Policies). Where applicable, authors should refer to any relevant reporting standards materials in this form.

For all that apply, please note **where in the article** the information is provided. Please note that we also collect information about data availability and ethics in the submission form.

Materials:

Newly created materials	Indicate where provided: section/figure legend	N/A
The manuscript includes a dedicated "materials availability statement" providing transparent disclosure about availability of newly created materials including details on how materials can be accessed and describing any restrictions on access.	N/A	N/A

Antibodies	Indicate where provided: section/figure legend	N/A
For commercial reagents, provide supplier name, catalogue number and RRID, if available.	All antibodies used in this study are listed in the main text (Table 2)	✓

DNA and RNA sequences	Indicate where provided: section/figure legend	N/A
Short novel DNA or RNA including primers, probes: Sequences should be included or deposited in a public repository.	All primers used in this study and their sequences are listed in the main text (Table 1)	✓

Cell materials	Indicate where provided: section/figure legend	N/A
Cell lines: Provide species information, strain. Provide accession number in repository OR supplier name, catalog number, clone number, OR RRID.	Human induced pluripotent stem cells (hiPSCs) used in this study are the HMGU1 cell line, derived from fibroblasts established from skin taken from normal foreskin from a neonatal male (ATCC	✓

	number CRL-2522, designation BJ). The HMGU1 cell line was a kind gift of Dr. Drukker. MTA approval was obtained from the Helmholtz Zentrum München (HMGU), Germany.	
Primary cultures: Provide species, strain, sex of origin, genetic modification status.	No primary cultures used in this study.	N/A

Experimental animals	Indicate where provided: section/figure legend	N/A
Laboratory animals or Model organisms: Provide species, strain, sex, age, genetic modification status. Provide accession number in repository OR supplier name, catalog number, clone number, OR RRID.	N/A	N/A
Animal observed in or captured from the field: Provide species, sex, and age where possible.	N/A	N/A

Plants and microbes	Indicate where provided: section/figure legend	N/A
Plants: provide species and strain, ecotype and cultivar where relevant, unique accession number if available, and source (including location for collected wild specimens).	N/A	N/A
Microbes: provide species and strain, unique accession number if available, and source.	N/A	N/A

Human research participants	Indicate where provided: section/figure legend) or state if these demographics were not collected	N/A
If collected and within the bounds of privacy constraints report on age, sex, gender and ethnicity for all study participants.	N/A	N/A

Design:

Study protocol	Indicate where provided: section/figure legend	N/A
If the study protocol has been pre-registered, provide DOI. For clinical trials, provide the trial registration number OR cite DOI.	N/A	N/A

**Laboratory protocol**	**Indicate where provided: section/figure legend**	**N/A**
Provide DOI OR other citation details if detailed step-by-step protocols are available.	All protocols are described in the section “Materials and methods”. For the immunostaining protocol, a more detailed version including all ordering codes has been previously published (doi: 10.21769/BioProtoc.3868).	✓

**Experimental study design (statistics details) ***		
**For in vivo studies: State whether and how the following have been done**	**Indicate where provided: section/figure legend. If it could have been done, but was not, write “not done”**	**N/A**
Sample size determination	N/A	N/A
Randomisation	N/A	N/A
Blinding	N/A	N/A
Inclusion/exclusion criteria	N/A	N/A

**Sample definition and in-laboratory replication**	**Indicate where provided: section/figure legend**	**N/A**
State number of times the experiment was replicated in the laboratory.	Experiments were replicated at least 3 times, unless otherwise stated in figure legends. Sample sizes are indicated in figure legends for each experiment. In graphs, samples typically represent technical replicates (example: measurement of intensity on an organoid section) obtained from at least 3 biological replicates (meaning 3 individual organoids) obtained from 2 distinct batches (meaning 2 independent culture experiments), unless otherwise stated. Refer to figure legends for exact number of replicates for each experiment.	✓
Define whether data describe technical or biological replicates.	Typically, data in graphs describe 8-10 technical replicates (distinct	✓

	sections) from 3-5 biological replicates (distinct organoids) obtained from 1-3 batches (independent experiments). For exact number for each quantification, please refer to figure legends. For MEA recordings, data were obtained from three distinct batches (each including 2 to 4 organoids on the MEA chip active surface). For single cell RNA sequencing, 2 distinct batches were pooled after cell dissociation, each batch consisting of 2 to 3 organoids. For further details, please refer to Figure Legends and to the Materials and Methods section of the paper.	

**Ethics**	**Indicate where provided: section/submission form**	**N/A**
Studies involving human participants: State details of authority granting ethics approval (IRB or equivalent committee(s), provide reference number for approval.	N/A	N/A
Studies involving experimental animals: State details of authority granting ethics approval (IRB or equivalent committee(s), provide reference number for approval.	N/A	N/A
Studies involving specimen and field samples: State if relevant permits obtained, provide details of authority approving study; if none were required, explain why.	N/A	N/A

**Dual Use Research of Concern (DURC)**	**Indicate where provided: section/submission form**	**N/A**
If study is subject to dual use research of concern regulations, state the authority granting approval and reference number for the regulatory approval.	N/A	N/A

Analysis:

**Attrition**	**Indicate where provided: section/figure legend**	**N/A**

Describe whether exclusion criteria were pre-established. Report if sample or data points were omitted from analysis. If yes, report if this was due to attrition or intentional exclusion and provide justification.	All data generated or analyzed are included in the figures. Exclusion criteria were applied for organoid section immunostainings and pixel intensity quantification: organoid sections with damaged histology were excluded from any further analysis/processing; the inner necrotic core of the organoid, when present, was excluded from counting/ quantification, as it only contained dead cells and debris.	✓

**Statistics**	**Indicate where provided: section/figure legend**	**N/A**
Describe statistical tests used and justify choice of tests.	Data were analyzed using the Mann–Whitney U-test or two-tailed Student's t-test (when comparing two data groups), or by two-way analysis of variance (ANOVA) for comparison of three or more groups. Statistical significance was set as follows: *$P<0.05$; **$P<0.01$; ***$P<0.001$. Please refer to the section Materials and Methods for a detailed description of the statistical analysis of single cell RNA sequencing results.	✓

**Data availability**	**Indicate where provided: section/submission form**	**N/A**
For newly created and reused datasets, the manuscript includes a data availability statement that provides details for access (or notes restrictions on access).	All data associated with this study are present in the paper or the Supplementary Materials.	✓
When newly created datasets are publicly available, provide accession number in repository OR DOI and licensing details where available.	N/A	N/A
If reused data is publicly available provide accession number in repository OR DOI, OR URL, OR citation.	N/A	N/A

**Code availability**	**Indicate where provided: section/figure legend**	**N/A**

For any computer code/software/mathematical algorithms essential for replicating the main findings of the study, whether newly generated or re-used, the manuscript includes a data availability statement that provides details for access or notes restrictions.	N/A	N/A
Where newly generated code is publicly available, provide accession number in repository, OR DOI OR URL and licensing details where available. State any restrictions on code availability or accessibility.	N/A	N/A
If reused code is publicly available provide accession number in repository OR DOI OR URL, OR citation.	N/A	N/A

Reporting:

The MDAR framework recommends adoption of discipline-specific guidelines, established and endorsed through community initiatives.

**Adherence to community standards**	**Indicate where provided: section/figure legend**	**N/A**
State if relevant guidelines (e.g., ICMJE, MIBBI, ARRIVE, STRANGE) have been followed, and whether a checklist (e.g., CONSORT, PRISMA, ARRIVE) is provided with the manuscript.	N/A	N/A

---

* We provide the following guidance regarding transparent reporting and statistics; we also refer authors to Ten common statistical mistakes to watch out for when writing or reviewing a manuscript.

Sample-size estimation

- You should state whether an appropriate sample size was computed when the study was being designed
- You should state the statistical method of sample size computation and any required assumptions
- If no explicit power analysis was used, you should describe how you decided what sample (replicate) size (number) to use

Replicates

- You should report how often each experiment was performed
- You should include a definition of biological versus technical replication
- The data obtained should be provided and sufficient information should be provided to indicate the number of independent biological and/or technical replicates
- If you encountered any outliers, you should describe how these were handled
- Criteria for exclusion/inclusion of data should be clearly stated
- High-throughput sequence data should be uploaded before submission, with a private link for reviewers provided (these are available from both GEO and ArrayExpress)

Statistical reporting

- Statistical analysis methods should be described and justified
- Raw data should be presented in figures whenever informative to do so (typically when N per group

is less than 10)

- For each experiment, you should identify the statistical tests used, exact values of N, definitions of center, methods of multiple test correction, and dispersion and precision measures (e.g., mean, median, SD, SEM, confidence intervals; and, for the major substantive results, a measure of effect size (e.g., Pearson's r, Cohen's d)
- Report exact p-values wherever possible alongside the summary statistics and 95% confidence intervals. These should be reported for all key questions and not only when the p-value is less than 0.05.

Group allocation

- Indicate how samples were allocated into experimental groups (in the case of clinical studies, please specify allocation to treatment method); if randomization was used, please also state if restricted randomization was applied
- Indicate if masking was used during group allocation, data collection and/or data analysis
